# Lysophosphatidylcholines Enriched with *cis* and *trans* Palmitoleic Acid Regulate Insulin Secretion via GPR119 Receptor

**DOI:** 10.1021/acsmedchemlett.3c00263

**Published:** 2024-01-09

**Authors:** Marcin Szustak, Eliza Korkus, Rafal Madaj, Arkadiusz Chworos, Grzegorz Dąbrowski, Sylwester Czaplicki, Erfan Tabandeh, Gabriela Maciejewska, Maria Koziołkiewicz, Iwona Konopka, Anna Gliszczyńska, Edyta Gendaszewska-Darmach

**Affiliations:** † Faculty of Biotechnology and Food Sciences, Institute of Molecular and Industrial Biotechnology, Lodz University of Technology, Stefanowskiego 2/22, 90-537 Lodz, Poland; ‡ Division of Bioorganic Chemistry Centre of Molecular and Macromolecular Studies, Polish Academy of Sciences, Sienkiewicza, 112, 90-363 Lodz, Poland; § Faculty of Food Sciences, Chair of Plant Food Chemistry and Processing, University of Warmia and Mazury in Olsztyn, Pl. Cieszyński 1, 10-957 Olsztyn, Poland; ∥ Institute of Evolutionary Biology, Faculty of Biology, Biological and Chemical Research Centre, University of Warsaw, Żwirki i Wigury 101, 02-089 Warsaw, Poland; ⊥ Central Laboatory of the Instrumental Analysis, Wroclaw University of Technology, Wybrzeze Wyspianskiego 27, Wroclaw 50-370, Poland; # Department of Food Chemistry and Biocatalysis, Wroclaw University of Environmental and Life Sciences, Norwida 25, 50-375 Wrocław, Poland

**Keywords:** Lysophosphatidylcholine, Palmitoleic acid, Diabetes, GPCR

## Abstract

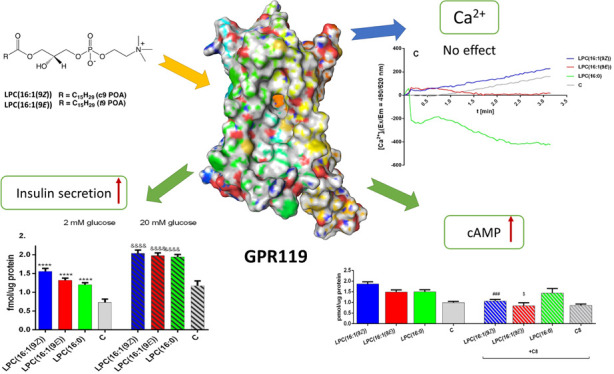

Among lipids, lysophosphatidylcholines (LPCs) with various fatty acyl chains have been identified as potential agonists of G protein-coupled receptors (GPCRs). Recently, targeting GPCRs has been switched to diabetes and obesity. Concomitantly, our last findings indicate the insulin secretagogue properties of *cis* and *trans* palmitoleic acid (16:1, n-7) resulting from GPCR activation, however, associated with different signaling pathways. We here report the synthesis of LPCs bearing two geometrical isomers of palmitoleic acids and investigation of their impact on human pancreatic β cells viability, insulin secretion, and activation of four GPCRs previously demonstrated to be targeted by free fatty acids and LPCs. Moreover, molecular modeling was exploited to investigate the probable binding sites of tested ligands and calculate their affinity toward GPR40, GPR55, GPR119, and GPR120 receptors.

Studies on lysophosphatidylcholines (LPCs) indicate their important role in the metabolism of glucose and lipids, broadening our knowledge of some disease mechanisms such as diabetes.^1,2^ LPCs, apart from their nonspecific membrane interactions, act as ligands of G protein-coupled receptors (GPCRs) GPR55^3^ and GPR119^1,2,4^ causing a mobilization of intracellular calcium, cyclic AMP (cAMP) synthesis, and an increase in insulin secretion. The structural geometry of LPC is a critical factor for the final activity and it depends mainly on the saturation and length of the acyl chain.^5^

Recently published data shed new light on GPR119 activation and its natural ligands. Xu et al. presented for the very first time cryo-EM structures of human GPR119-Gs complex with LPCs and APD668, a type 2 diabetes clinical drug candidate.^4^ They found that LPC occupies GPR119 without the ligand added exogenously. Mass spectrometry of the analyzed complex showed the most abundant LPC molecules with 18:1, 18:0, 16:1, and 20:0 acyl chains. LPCs were docked into the top half of GPR119 into an extended shallow pocket formed by all seven transmembrane helices and ECL2. The binding pocket was composed of a hydrophilic pocket responsible for the polar contact between the receptor and the LPC head and a hydrophobic pocket that locates the acyl group during receptor activation. Meanwhile, Qian et al. also utilized cryo-EM to reveal the unique structure of the binding pocket in the GPR119-Gs complex with AR231453 and MBX-2982 synthetic agonists. The authors divided the pocket into three main elements with different roles: extracellular cavity, stacking gate, and activation cavity.^6^

LPC with the acyl tail of a monounsaturated palmitoleic acid (POA, 16:1) was identified as one of the most abundant ligands for GPR119.^4^ POA is classified as a representative of the omega-7 fatty acid group.^7^ The common in nature, bent (9*Z*)-hexadecenoic acid (C16:1(9*Z*)) isomer comes predominantly from endogenous sources; however, some plant and animal-based foods contain trace amounts. On the other hand, straight (9*E*)-hexadecenoic acid (C16:1(9*E*)) originates mainly from dairy products.^8^ C16:1(9*Z*) itself affects glucose metabolism by stimulating insulin secretion and reducing insulin resistance.^9,10^

Our recent findings confirm the insulin secretagogue properties of the C16:1(9*Z*) isomer and show that this biological function depends on the activation of GPR40, GPR55, GPR119, and GPR120 receptors.^11^ All these GPCRs are considered potential targets for the pharmacological management of diabetes.^12−15^ The C16:1(9*E*) isomer also augments insulin release from pancreatic β cells but signals not only through G_q_ as observed with the endogenous lipid ligands, including C16:1(9*Z*), but also through G_s_.^16^

If the difference in acyl group conformation in POA affects receptor activation in the context of GPCR signaling, then we decided to investigate whether the double bond configuration of the acyl tail also influences the mode of LPC binding with GPR40, GPR55, GPR119, and GPR120. Although Xu et al. identified LPC(16:1) (isomer not specified) inside the GPR119 ligand pocket, they did not investigate its role in receptor activation thoroughly. Therefore, we first applied molecular modeling to identify predicted interactions of LPCs containing two isomers of POA, namely, LPC(16:1(9*Z*)) or LPC(16:1(9*E*)). The results prompted us further to synthesize LPCs containing in the *sn*-1 position pure isomers of hexadecenoic acid (16:1) (9*Z*) and (9*E*), respectively, and investigate their activities in pancreatic EndoC-βH1 cells.

A computer-aided drug design protocol with membrane generation, molecular docking, and molecular dynamics with trajectory post processing was used. High stability of receptor structures was observed throughout the simulations, as both Cα RMSD and radius of gyration remained stable with small shifts deriving from α-helices not embedded inside the membrane (Figures S1 and S2). Ligands tended to drift near the initial position (Figure S3). Despite notable fluctuations, LPC(16:1(9*E*)) seemed to be buried in the highest degree in the case of receptors GPR119 and GPR55, while LPC(16:1(9*Z*)) was completely covered by GPR40s residues (Figure S4). Binding energies indicate that solely GPR119 can bind LPCs with high affinity, with a preference toward LPC(16:1(9*E*)) (Table 1). Moreover, LPC(16:1(9*Z*)) could interact with GPR55 but with a weaker energy than GPR119. The energies of the complexes with GPR40 and GPR120 demonstrated a lack of binding (Table 1, Figures S6–S8). An interesting result could be observed in the LPC ligands bound to GPR119. GPR119 binding pocket is open to the extracellular side and leads straight into the cellular membrane where the bottom of this structure is located (Figure S5). In the case of both analyzed LPCs, the hydrophobic tail is located deep in the binding pocket, whereas the polar side of LPC stays on the polar entrance. Polar part of LPC(16:1(9*Z*)) interact with Gln65, Gln154, Cys155, and Ser156 (Figure 1). Similarly, the GPR119 polar pocket also binds LPC(16:1(9*E*)), but it also attracts Met1, Glu2, and Arg262 from the surrounding loops. Consequently, this ligand is more tightly connected to the receptor (Figure 1). Some differences are also observed inside the active site of the binding pocket which is connected with different geometry of two isomers. LPC(16:1(9*Z*)) bends in the middle and interacts mainly with Val85, Thr86, Ala89, Phe241, Leu242, and Trp265. Admittedly, Trp238, a toggle switch of the receptor,^6^ is also located in the ligand neighborhood, but its activation by double bond interaction (π–π) could be difficult because of the distance between them. LPC(16:1(9*E*)) goes straight through the GPR119 bidding pocket, which is connected with this molecule geometry. It creates interactions with Thr86, Ala90, Leu94, Leu169, Phe174, Trp238, Phe241, and Trp265. In this case, a double bond of LPC(16:1(9*E*)) is located in front of Trp238 that easily could activate the receptor. Indicated amino acid residues were exchanged with Ala during *in silico* point mutagenesis with the ICM-Pro software.^17^ Phe157, Trp238, and Phe241 were confirmed to be crucial for binding of both isomers (Figure S9). Xu et al. identified the long binding pocket with a hydrophilic entrance from the extracellular side and a hydrophobic interior when analyzing that LPC(18:1) bound to GPR119. Polar residues such as Glu261 and Arg262 created interactions with choline and the phosphate moiety, whereas Ser165 and Gln65 formed hydrogen bonds with the LPC head. Nonpolar part of LPC went deep inside the pocket to create interactions with Ala89, Ala90, Val93, Leu94, Ile146, Phe157, Phe158, Trp238, and Trp265. Mutagenesis data clearly showed the importance of the above-mentioned residues in the LPC biding and signal transduction process.^4^

**Table 1:** Results of Molecular Docking and Dynamics with Final Energy of the Binding of LPC(16:1(9*Z*)) and LPC(16:1(9*E*)) into the Analyzed GPCRs

receptor	ligand	docking binding energy [kcal/mol]	MD relative energy of binding [kcal/mol]	ligand RMSD [A]	SASA [A^2^]
GPR40	LPC(16:1(9*Z*))	–2 ± 0.4	–12 ± 10	3.4 ± 0.5	–0.4 ± 40.1
	LPC(16:1(9*E*))	–2 ± 0.3	4 ± 11	2.8 ± 0.3	111.9 ± 68.7
GPR55	LPC(16:1(9*Z*))	–3.5 ± 2.2	–34 ± 13	2.8 ± 0.3	111.9 ± 68.7
	LPC(16:1(9*E*))	–2.4 ± 2	–19 ± 12	2.7 ± 0.7	52.4 ± 40.9
GPR119	LPC(16:1(9*Z*))	–3.5 ± 2.4	–41 ± 11	3.3 ± 0.5	110.6 ± 75.4
	LPC(16:1(9*E*))	–2 ± 1.8	–45 ± 19	4.0 ± 0.5	54.6 ± 63.2
GPR120	LPC(16:1(9*Z*))	–1.3 ± 2	–19 ± 15	3.2 ± 0.5	139.4 ± 105.9
	LPC(16:1(9*E*))	–2.9 ± 3.7	–12 ± 12	3.9 ± 0.6	153.4 ± 102.7

**Figure 1:**
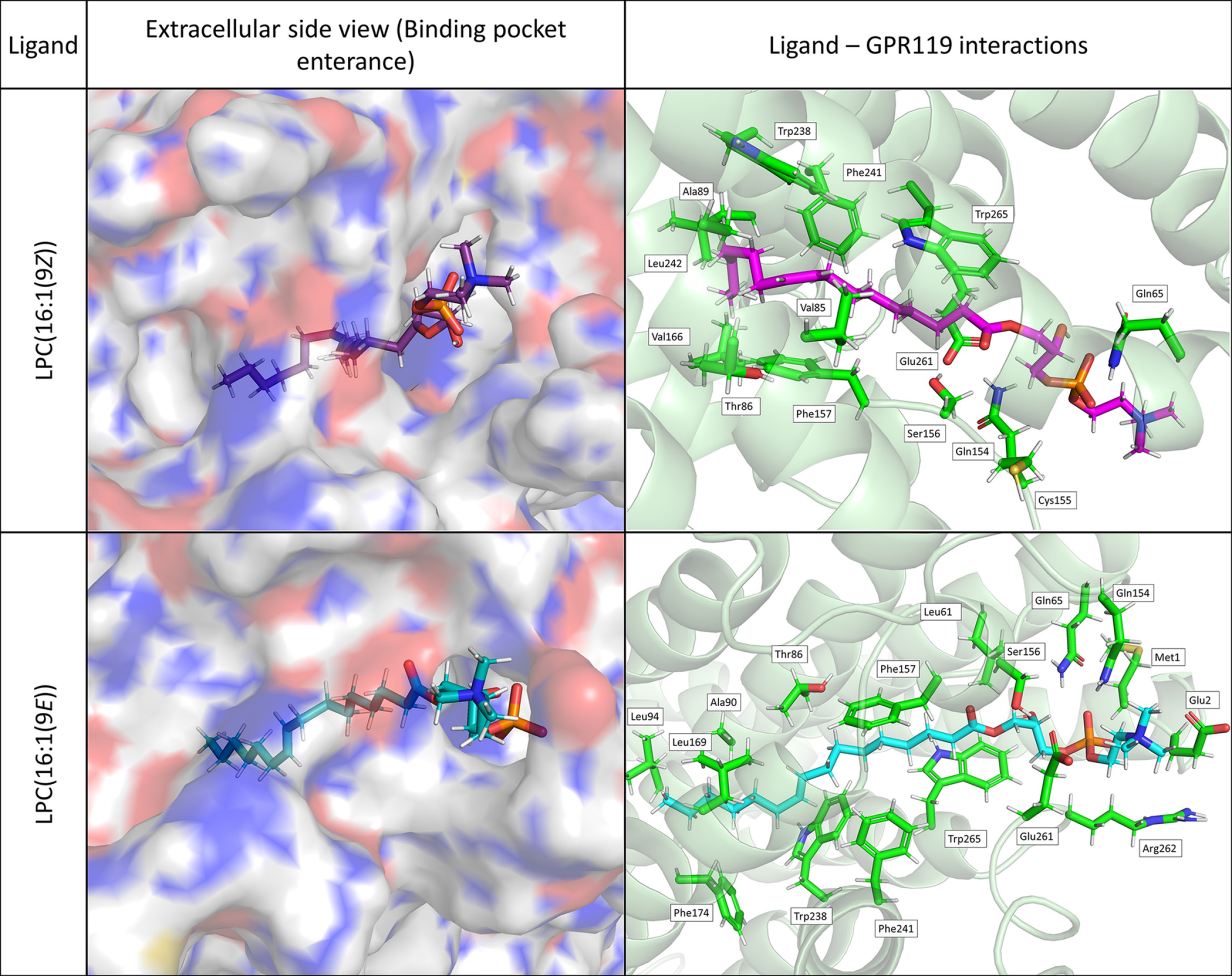
Docking poses of LPC(16:1(9Z)) and LPC(16:1(9E)) in the binding pocket of GPR119

Qian et al. also prepared a cryo-EM-based model of GPR119 combined with AR231453 and MBX-2982 agonists. They indicated the importance of Trp238 as a toggle switch of the receptor. Also, mutagenesis analyses confirmed a key role of residues located in the activation site, such as Thr86, Ala89, Val93, Leu94, Leu169, and Leu242^6^ which are also important in our models. The authors also prepared molecular docking of the GPR119 endogenous agonist oleoylethanolamide (OEA). OEA is located similarly to AR231453 and MBX-2982 and any mutations of Thr86, Val93, Leu94, Ile136, Leu169, Phe174, or Phe241 caused significant activity loss. Moreover, the double bond of the OEA acyl chain located opposite Trp238 allowed it to form π–π interactions and activate the receptor.^6^ Our model did not differ significantly from those described above. Concluding, in a silico study, we demonstrated that LPC(16:1(9*Z*)) and LPC(16:1(9*E*)) are not favored ligands in all but one GPCR receptor: GPR119. Both LPCs penetrate the activation cavity from the extracellular side and are located in the Trp238 neighborhood, a toggle trigger for GPR119. LPC(16:1(9*E*)) creates stronger interaction than LPC(16:1(9*Z*)), binds more polar residues at the entrance of the binding pocket, and locates closer to the activation site of GPR119.

To confirm the ability of LPC(16:1(9*Z*)) and LPC(16:1(9*E*)) to activate the GPR119 receptor we synthesized those molecules (Scheme 1). LPCs (**2a**, **2b**) with C16:1(9*Z*) and C16:1(9*E*) at the *sn*-1 position were synthesized from *sn*-glycerophosphocholine (GPC) according to the procedure that we previously reported.^18^ First, GPC was treated with dibutyltin oxide (DBTO) transformed into cyclic stannylene ketal, which was subsequently selectively acetylated with chlorides of *cis* and *trans* POA (Scheme 1). The resulting acetal was subjected next to a reaction with triethylamine (TEA) and appropriate chlorides of fatty acids, which were previously obtained *in situ*. The 1-palmitoleoyl-2-hydroxy-*sn*-glycero-3-phosphocholine (LPC(16:1(9*Z*)) and 1-palmitelaidiceoyl- 2-hydroxy-*sn*-glycero-3-phosphocholine (LPC(16:1(9*E*)) were synthesized in high 49% and 48% yields, respectively. The products were next fully characterized by ^1^H, ^13^C, ^31^P NMR, and high-resolution mass spectrometry (HRMS). Signals characteristic for the 9*Z* and 9*E* olefinic protons in the chain of the acyl residue of LPCs were identified in the ^1^H NMR at 5.12–5.13 ppm, whereas signals from the −N^+^(CH_3_)_3_ groups were detected at 3.01 and 3.03 ppm, respectively. The multiples of protons H-2′ in the range 3.63–3.92 ppm proved that the *sn*-2 position was nonesterified in obtained LPCs. The carbon atoms of the double bonds in LPC(16:1(9*Z*)) and LPC(16:1(9*E*)) gave signals in the ^13^C NMR spectra in the range of 129.59–130.39 ppm. On the spectra from ESI-MS we observed a molecular ion [M]^+^ at *m*/*z* 494.32, which is characteristic for these compounds. Detailed assignments are given in the Supporting Information, including the experimental section from the chemical part of this work.

**Scheme 1:**
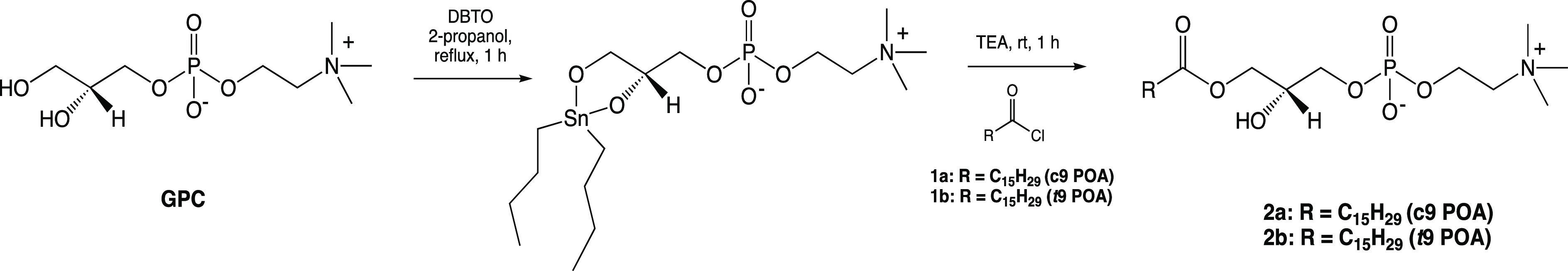
Synthetic Scheme of 1-Acyl-2-hydroxy-*sn*-glycero-3-phosphocholines **2a** and **2b**

Subsequently, the human pancreatic β cell line, EndoC-βH1, was used as a model to confirm molecular modeling showing preferential binding to GPR119 and to determine the impact of LPC(16:1(9*Z*)) and LPC(16:1(9*E*)) on glucose-stimulated insulin secretion. We have previously demonstrated the expression of *GPR40*, *GPR55*, *GPR119*, and *GPR120* in EndoC-βH1 cells.^16^ Since *trans* fatty acids resemble the linear form of saturated fat, we also included LPC(16:0) in biological investigations. LPC(16:0) was shown previously to activate GPR119 in transfected cell lines^4,19^ and pancreatic β cell lines.^20^ Also, molecular modeling of GPR119 with LPC(16:0) clearly showed the strong affinity of this ligand to the receptor with a total electrostatic interaction energy of about -30 kcal/mol. We also indicated Arg262 as the most important for LPC(16:0) binding into the receptor pocket, but unfortunately, we could not show any engaging nonpolar interaction in the active site of the receptor. However, the use of a model generated with the use of the TASSER library of the Zhang lab which mainly focused on the entrance gate of the binding pocket could be the reason for some inaccuracies.^20^ Our present models are based on CryoEM structures, which makes them more reliable.

As LPCs may be harmful to human cells, cell viability after 24, 48, and 72 h of incubation with LPC(16:1(9*Z*)), LPC(16:1(9*E*)), and LPC(16:0) was determined first (Figure 2). In general, the highest the concentration of the tested compound the lower cell survivability was observed. LPC(16:1(9*Z*)) was not toxic at concentrations up to 10 μM under all treatment times. In contrast, LPC(16:1(9*E*)) appeared to be much more harmful to cells, and only 1 μM concentration did not reduce cell viability. Long-term culture with 5 μM or higher concentration of LPC(16:1(9*E*)) for 48 and 72 h eliminated almost all living cells. LPC(16:0) seemed to be less toxic than LPC(16:1(9*E*)) despite a similar molecule geometry. No significant reduction of viability was observed for LPC(16:1(9*Z*)) and LPC(16:1(9*E*)) were then evaluated alongside LPC(16:0) in a GSIS assay. As far as potential antidiabetic activity is concerned, LPC(14:0), LPC(16:0), LPC(18:0), and LPC(18:1) were tested so far as insulin secretagogues.^2,21,22^ 1 μM LPCs under study did not potentiate insulin secretion (data not shown). The lowest effective concentration of 5 μM improved the responsiveness of the EndoC-βH1 cells (Figure 3). Although the toxicity of LPC(16:1(9*E*)) was observed in long-term experiments, we decided to increase the amount of added LPCs up to 5 μM since insulin secretion is a short-term study (the analysis is performed after 1.5 h of incubation). To control the permeability of the cell membrane and to ensure that insulin release does not occur as a result of membrane disruption we also measured the level of propidium iodide uptake by cells. None of the tested LPCs induced membrane permeabilization, even at concentrations of 10 μM during short-term incubation (Figure 2F).

**Figure 2:**
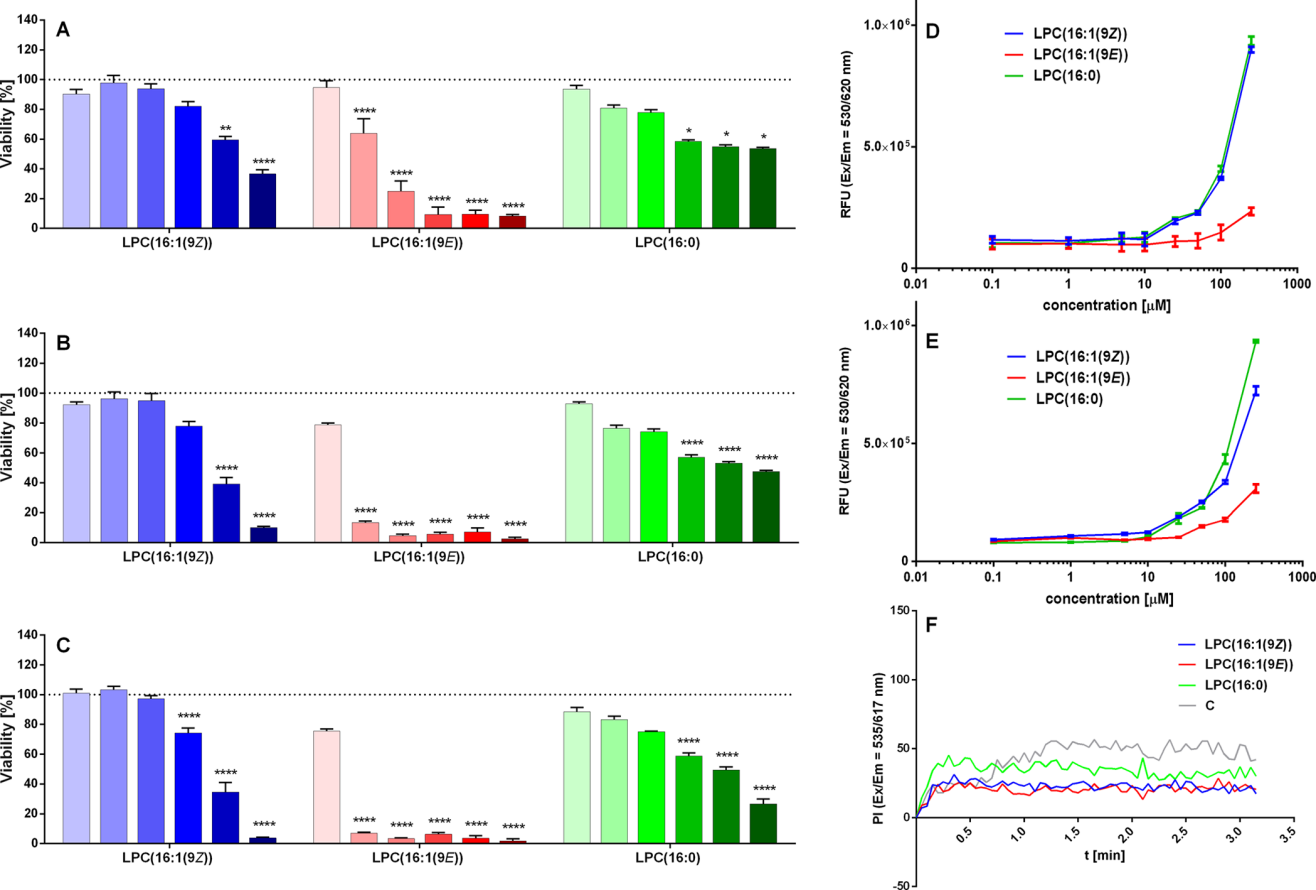
Viability of the human pancreatic EndoC-βH1 cells after 24 (A), 48 (B), and 72 h (C) of treatment with LPCs in the range from 1 to 100 μM (the increasing intensity of color in the bars corresponds to 1, 5, 10, 25, 50, and 100 μM). Critical micelle concentration presented as a Nile Red fluorescence intensity on the logarithmic concentration of LPC(16:1, n-7) isomers and LPC(16:0) in DMEM culture medium (D) and Ca5 buffer (E). PI intercalation in the presence of LPC(16:1, n-7) isomers and LPC(16:0) in Ca5 buffer with 20 mM glucose vs compound solvent control assessed at 5 μM concentration. The results are presented as the real-time kinetics of [PI] changes inside the cell during 3 min-time monitoring (F). Data represent the means ± SEM from at least 3 independent experiments. ****p < 0.0001, **p < 0.01, *p < 0.05 vs control.

**Figure 3:**
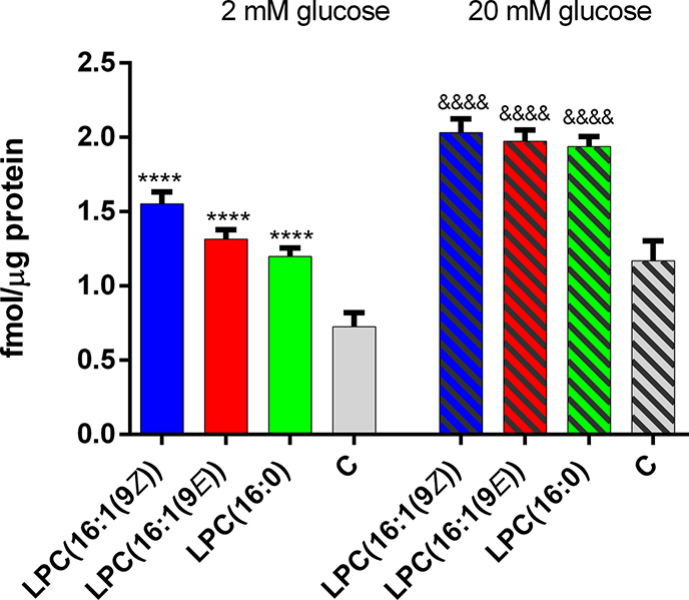
Effect of tested LPCs applied at 5 μM on insulin secretion. Data represent the means ± SEM from at least 3 independent experiments. ****p < 0.0001 vs 2 mM glucose control; ^&&&&^*p* < 0.0001 vs 20 mM glucose control.

All tested compounds stimulated the secretion of insulin at a similar level at both 2 and 20 mM glucose content (Figure 3). To find out if tested LPCs affect insulin secretion via one of the analyzed GPCR receptors, a series of experiments with antagonists was performed. In general, the application of GPR40, GPR55, and GPR120 antagonists to the EndoC-βH1 cells did not significantly affect insulin secretion evoked by all tested LPCs (Figure 4A). A small reduction of secreted insulin was observed in the experiment with the GPR55 antagonist when LPC(16:1(9*E*)) and LPC(16:1(9*Z*)) was present (Figure 4). In contrast, the most spectacular reduction was observed when the GPR119 antagonist was added. C8 reduced insulin release from 1.97 to 1.34 fmol/μg protein in the presence of LPC(16:1(9*E*)) and from 2.03 to 1.47 fmol/μg protein where LPC(16:1(9*Z*)) were used. Also, LPC(16:0) stimulation was slightly reduced after pretreatment with the GPR119 antagonist (Figure 4A). Antagonists themselves did not affect insulin secretion.

**Figure 4:**
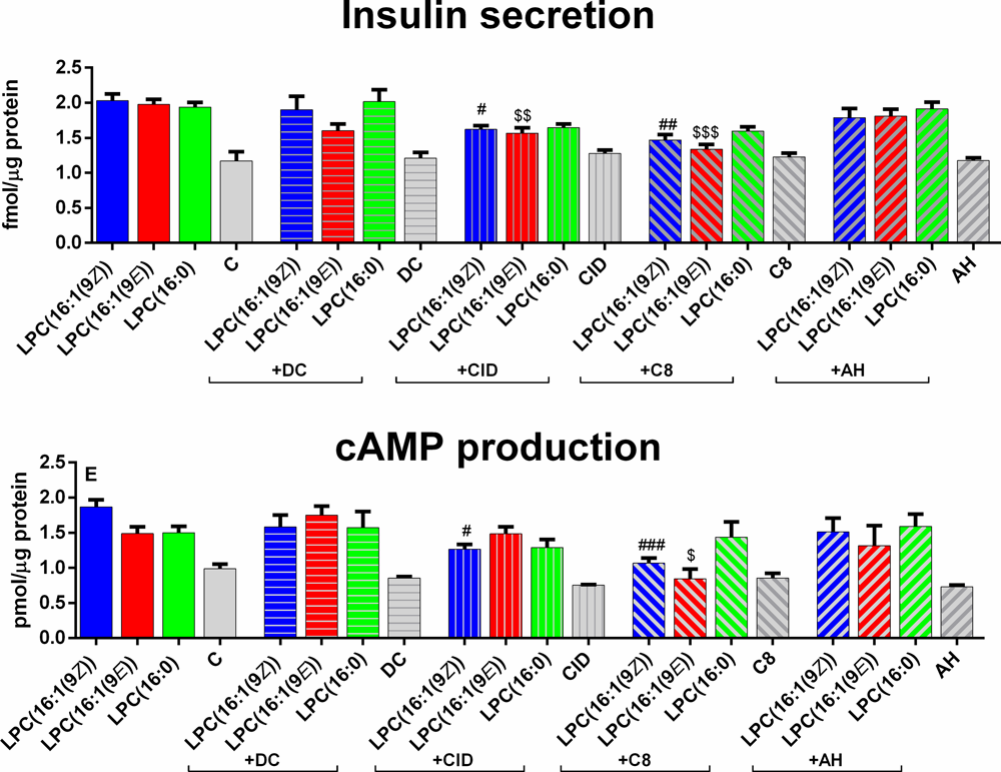
Effect of 5 μM tested LPCs on insulin secretion and cAMP production in EndoC-βH1 cells. DC, CID, C8 and AH represents antagonists for GPR40, GPR55, GPR119 and GPR120 consecutively. Experiments were performed under 20 mM glucose conditions. Data represent the means ± SEM from at least 3 independent experiments. ^###^*p* < 0.001 vs LPC(16:1(9*Z*)); ^##^*p* < 0.01 vs LPC(16:1(9*Z*)); ^#^*p* < 0.05 vs LPC(16:1(9*Z*)); ^$$$^*p* < 0.001 vs LPC(16:1(9*E*)); ^$^*p* < 0.05 vs LPC(16:1(9*E*)).

After ligand binding GPCRs undergo conformational changes which lead to the transfer signal to cytosolic G proteins and β-arrestin.^23^ Long-chain fatty acids activate the G_q/11_ pathway after binding GPR40 and GPR120, while the main signaling pathway associated with GPR119 is related to the activation of G_s_ in response to endogenous lipids. GPR55 has been shown to utilize G_q_, G_12/13_, and G_s_ for signal transduction.^24−27^ G_q_ is responsible for phospholipase C activation leading to increased intracellular calcium concentration during GSIS. On the other hand, G_s_ promotes cAMP synthesis by adenylyl cyclase activation and as a result, insulin release is observed.^24^ To find out which intracellular pathways were stimulated by tested LPCs in pancreatic EndoC-βH1 cells, intracellular calcium mobilization and cAMP production were determined.

The addition of LPCs to EndoC-βH1 cells did not cause any significant growth in intracellular calcium ion mobilization (Figure 5). All tested compounds gave a plot close to zero or below control. Only LPC(16:1(9*Z*)) showed a very small increase. On the other hand, exposure of EndoC-βH1 cells to all tested LPCs resulted in prominent stimulatory effects on the production of cellular cAMP with the highest value observed for LPC(16:1(9*Z*)) (1.87 pg/μg protein) (Figure 4B). Antagonists of GPR40 and GPR120 did not affect cAMP production significantly (Figure 4B), whereas GPR119 antagonist inhibited LPC(16:1(9*Z*))- and LPC(16:1(9*E*))-induced cAMP rise by 0.80 pmol/μg protein and 0.65 pmol/μg protein, respective. On the other hand, cAMP production stimulated by LPC(16:0) was not inhibited by any of the used antagonists. Chu et al. showed that AR231453, the agonist of GPR119, caused cAMP accumulation in GLUTag cells.^28^ Three years later, Hansen et al. demonstrated that GPR119 activated by 2-monoacylglycerols also led to elevated cAMP level.^19^ The results obtained in this study confirm the previous observations that GPR119 is mainly coupled with the G_s_ protein activating adenylyl cyclase.

**Figure 5:**
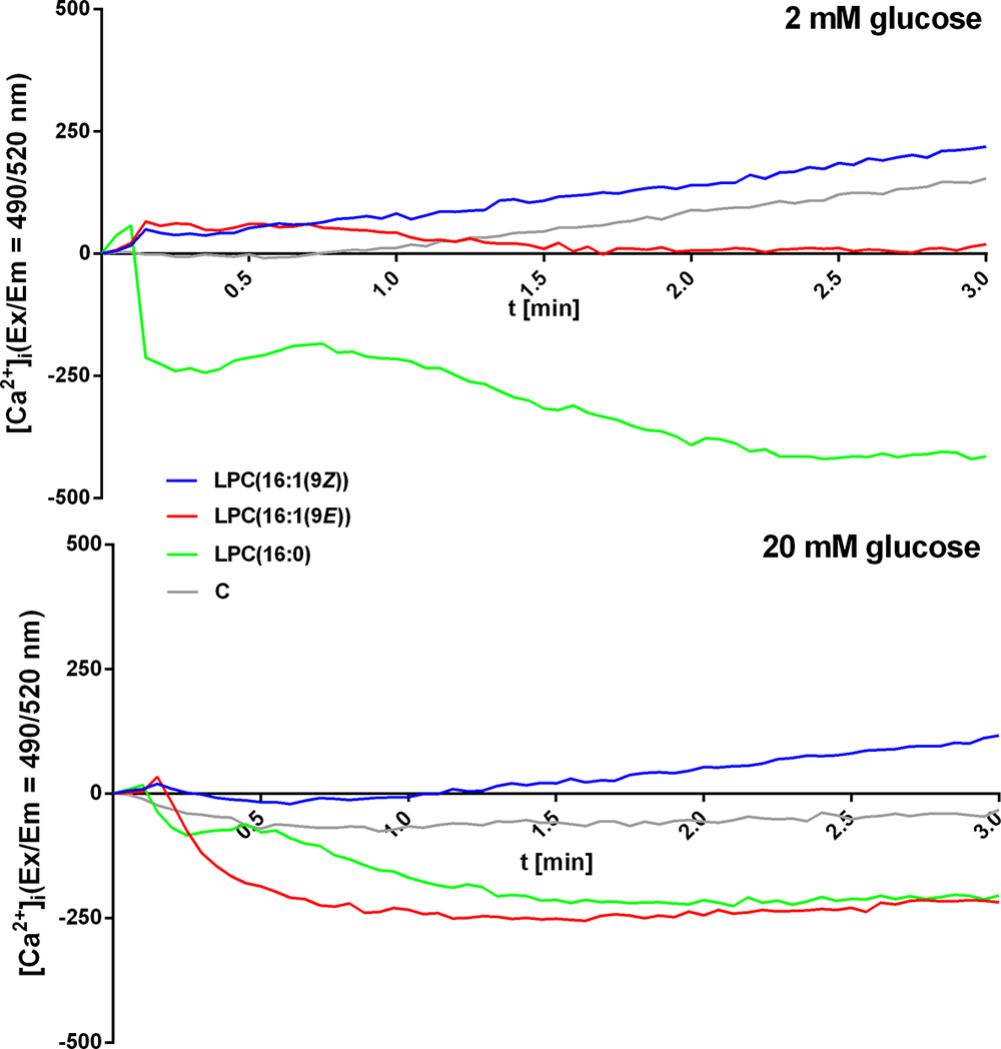
Effect of 5 μM tested LPCs on intracellular Ca^2+^ mobilization in EndoC-βH1 cells. The results are presented as the real-time kinetics of [Ca^2+^]i changes inside the cell during 3 min-time monitoring. Data represent the means ± SEM from at least 3 independent experiments.

While GPR119 activates the pathway related to the accumulation of cAMP, the mechanism of action of GPR55 seems to be more complicated and dependent on the isomer of palmitoleic acid. The GPR55 antagonist caused the reduction of cAMP when LPC(16:1(9*Z*)) was applied. LPC(16:1(9Z)) also slightly increased the amount of intracellular calcium ions. At the same time, the cAMP growth induced by LPC(16:1(9*E*)) was not GPR55-dependent suggesting that this isomer acts mainly via activation of GPR119. Moreover, we also found out that AR231453, the prototypical potent and orally available GPR119 selective agonist, evoked insulin release at high glucose concentration, with 100 nm concentration being effective. At the same time, the lowest effective concentration of LPC(16:1(9Z)) was estimated as 1 μM. So, the potency of LPC(16:1(9Z)) is at least 10 times lower as compared with AR231453 in the applied EndoC-βH1 cellular model (Figure 6). To enhance our findings and prove that GPR119 was stimulated by tested LPCs, gene silencing targeting GPR119 was established (Table S2). Subsequently we confirmed insulin secretion reduction in cells transfected with siRNA against GPR119 which prove LPC(16:1) role as a ligand for this receptor (Figure S12).

**Figure 6:**
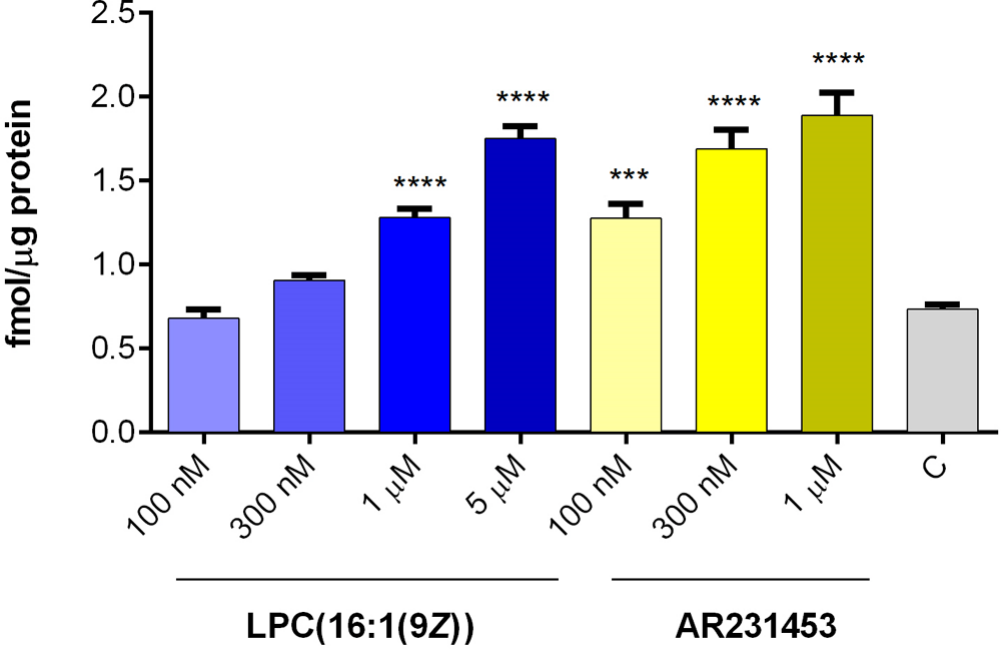
Effect of GPR119 agonist AR231453 and LPC(16:1(9*Z*)) on insulin secretion. Data represent the means ± SEM from at least 3 independent experiments. ****p < 0.0001 vs 20 mM glucose control.

Overall, LPC(16:1(9*Z*)), LPC(16:1(9*E*)), and LPC(16:0) increased GSIS with similar potency despite different cytotoxicities against pancreatic EndoC-βH1 cells. Insulin secretion was strongly inhibited by the presence of the GPR119 antagonist. The addition of the GPR55 antagonist showed a negligible effect while GPR40 and GPR120 antagonists had no effect at all.

To summarize, LPC(16:1(9*Z*)) stimulates GPR55 and GPR119 leading to the stimulation of the G_s_ cascade. Also, LPC(16:1(9*E*)) stimulates the G_s_ cascade via both receptors. Intracellular calcium remained stable during LPC addition and did not differ from control unstimulated cells significantly. This indicates no participation of the G_q_ cascade in insulin secretion stimulated by analyzed LPCs. All of these biological results strongly correlate with modeling calculations. LPC(16:1(9*Z*)) affects GPR55 and GPR119 with relative biding energy −34 and −41 kcal/mol, respectively, and −45 kcal/mol for binding LPC(16:1(9*E*)) to GPR119. Xu et al. presented that LPC(16:0) activates GPR119 in human embryonic kidney 293 cells, leading to cAMP accumulation. They also showed the presence of LPC(16:1) in GPR119-G_s_ complex.^4^ In our work, both LPC(16:1) isomers strongly affect GPR119 and lead to cAMP accumulation higher than that of their saturated counterpart.

The spatial structure of a molecule may have a critical influence on its function. Therefore, it is very important to study the biological activity of different isomers of the same compound to find out which one presents demand properties.^29^ To date, there are no data on biological research that compares the activity of different stereoisomers of fatty acids esterified in lysophospholipids and the very few where fatty acid stereoisomers alone are investigated. One example describes a conjugated linoleic acid (CLA) which is a mixture of different isomers of octadecadienoic acid C18:2. C18:2(*Z*9*E*11) and C18:2(*E*10*Z*12) are the most abundant.^30^ In general, CLA mixture provides beneficial properties preventing atherosclerosis or different types of cancer,^30,31^ but recent analyses with separated isomers show a difference in their activity. C18:2(*E*10*Z*12) reduces the activity of lipoprotein lipases and the level of intracellular glycerol and triacylglycerols, while C18:2(*Z*9*E*11) does not.^32^ In contrast, C18:2(*Z*9*E*11) inhibits tumor necrosis factor-α production and suppresses the generation of neurotoxic amyloid β but C18:2(*E*10*Z*12) does not.^33,34^ Moreover, diet supplementation with C18:2(*Z*9*E*11) results in the growth of LPC(C18:2(*Z*9*E*11)) content in neurons.^34^ Also, our previous findings show that both isomers C16:1(*E*9) and C16:1(*Z*9) enhance glucose-stimulated insulin secretion, but they achieve it with different intracellular signaling pathways.^16^

In conclusion, the results presented here are the very first that comprehensively show the biological effect, manifested as enhanced insulin secretion during glucose stimulation, of two different isomers of palmitoleic acid incorporated into LPC. We were able to indicate that LPC(16:1(9*Z*)) and LPC(16:1(9*E*)), despite the differences in cytotoxicity and CMC value, act as GPR55 and GPR119 ligands and they activate G_s_ cascade presented as cAMP accumulation. However, the activity of LPC(16:1(9*E*)) was completely abolished by the GPR119 antagonist in contrast with LPC(16:1(9*Z*). LPC(16:1(9*Z*)) also activates the GPR55 receptor more avidly. These biological results were confirmed by molecular modeling calculations.

## Supporting Information Available

Methodology and additional figures (PDF)

## Abbreviations

AH: AH7614 - GPR120 antagonist
C8: GPR119 antagonist
cAMP: cyclic adenosine monophosphate
CID: CID16020046 - GPR55 antagonist
CLA: conjugated linoleic acids
CMC: critical micelle concentration
LPC(16:1(9*Z*)): *cis*-palmitoleoyl-lysophosphatidylcholine
DC: DC260126 - GPR40 antagonist
GPCR: G protein-coupled receptors
GSIS: glucose-stimulated insulin secretion
LPC: lysophosphatidylcholine
PI: propidium iodide
POA: palmitoleic acid
LPC(16:0): palmitoyl-lysophosphatidylcholine
*R*_g_: radius of gyration
RMSD: root-mean square deviation
SASA: solvent-accessible surface area
tPOA: *trans* palmitoleic acid
LPC(16:1(9*E*)): *trans*-palmitoleoyl-lysophosphatidylcholine
