# Supplementary material for: Lysophosphatidylcholines Enriched with cis and trans Palmitoleic Acid Regulate Insulin Secretion via GPR119 Receptor
Source: ACS Med Chem Lett. 2024 Jan 9;15(2):197–204. doi: 10.1021/acsmedchemlett.3c00263 (PMC10860191; doi:10.1021/acsmedchemlett.3c00263)
Supplement: Supplementary file 1 — ml3c00263_si_001.pdf [file ml3c00263_si_001.pdf]

SUPPORTING INFORMATION

Lysophosphatidylcholines enriched with *cis* and *trans* palmitoleic acid regulate insulin secretion via GPR119 receptor

Marcin Szustak[a*‡], Eliza Korkus[a‡], Rafal Madaj[c,d], Arkadiusz Chworos[c] Grzegorz Dąbrowski[b], Sylwester Czaplicki[b], Erfan Tabandeh[a], Gabriela Maciejewska[e], Maria Koziołkiewicz[a], Iwona Konopka[b], Anna Gliszczyńska[f], Edyta Gendaszewska-Darmach[a]

[a]Faculty of Biotechnology and Food Sciences, Institute of Molecular and Industrial Biotechnology, Lodz University of Technology, Stefanowskiego 2/22, 90-537 Lodz, Poland
[b]Faculty of Food Sciences, Chair of Plant Food Chemistry and Processing, University of Warmia and Mazury in Olsztyn, Pl. Cieszyński 1, 10-957 Olsztyn, Poland
[c]Division of Bioorganic Chemistry Centre of Molecular and Macromolecular Studies, Polish Academy of Sciences, Sienkiewicza, 112, 90-363 Lodz, Poland
[d]Institute of Evolutionary Biology, Faculty of Biology, Biological and Chemical Research Centre, University of Warsaw, Żwirki i Wigury 101, 02-089, Warsaw, Poland
[e]Central Laboratory of the Instrumental Analysis, Wroclaw University of Technology, Wybrzeze Wyspianskiego 27, Wroclaw 50-370, Poland
[f]Department of Food Chemistry and Biocatalysis, Wroclaw University of Environmental and Life Sciences, Norwida 25, 50-375 Wrocław, Poland

Table of contents:

1. Molecular modeling methodology.
2. Fig S1. Root mean square deviation of Cα atoms.
3. Fig S2. Radius of gyration.
4. Fig S3. Solvent accessible surface area.
5. Fig S4. Root mean square deviation of ligand during simulations.
6. Fig S5. Structure of GPR119.
7. Fig S6. Most frequent position obtained through clustering LPC(16:1(9*Z*)) and LPC(16:1(9*E*)) inside the GPR40.
8. Fig S7. Most frequent position obtained through clustering LPC(16:1(9*Z*)) and LPC(16:1(9*E*)) inside the GPR55.
9. Fig S8. Most frequent position obtained through clustering LPC(16:1(9*Z*)) and LPC(16:1(9*E*)) inside the GPR120.
10. Mutagenesis *in sil*ico
11. Fig S9. Binding energy changes in mutants where amino acids located in receptor pocket are replaced with alanine.
12. LPC synthesis.
13. Fig S10. $_{1}$H and $_{13}$C NMR spectra and $_{31}$P NMR of 1-palmitoleoyl-2-hydroxy-sn-glycero-3-phosphocholine.
14. Fig S11. $_{1}$H and $_{13}$C NMR spectra and $_{31}$P NMR of 1-palmitelaidiceoyl-2-hydroxy-sn-glycero-3-phosphocholine.
15. Cell viability assay
16. Critical micelle concentration methodology
17. siRNA transfection
18. Calcium flux measurements

19. Glucose stimulated insulin secretion (GSIS)
20. siRNA transfection preceding GSIS
21. Fig. S12. Insulin secretion by EndoC-bH1 cells after 48 hours of transfection with siRNA
22. cAMP synthesis measurements
23. Safety statement
24. References

Molecular modeling

To keep the continuity of the methodology, all molecular docking, conventional molecular dynamics and trajectory postprocessing was performed using GNINA 1.0[1], AMBER software[2] with a protocol described previously[3], with the exception that ligands RESP charge fitting at a total charge of 0 site-oriented molecular docking performed after obtaining initial poses from blind docking and MMGBSA calculations with topology and trajectory stripped from the membrane after the calculations for increased accuracy. The structure of GPR40 and GPR119 receptors were downloaded from the protein data base (ID: 4PHU, 7XZ5 respectively) Receptors GPR55 and GPR120 were folded by AlphaFold2[4]. Then receptors were embedded into the membrane using CHARMM-GUI[5]. Visualization of protein-ligand complexes was performed with the use of PyMOL software with the help of PLIP interaction profiler[6].

Stability of structures during simulations was validated by a radius of gyration (Rg), root-mean square deviation (RMSD) of Cα atoms of residues employed in secondary structure formation, solvent-accessible surface area of ligand (SASA) as well as its RMSD throughout the simulations.

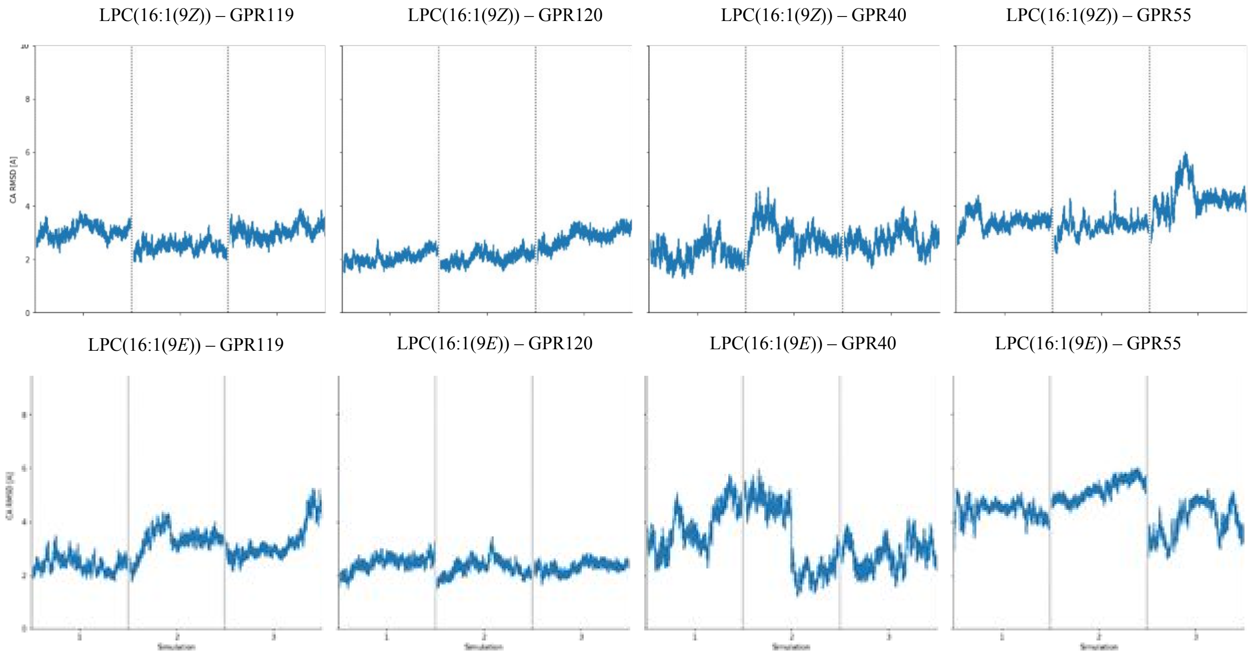

Fig S1. Root mean square deviation of Cα atoms contributing to secondary structure formation. Small shifts are observable during the displacement of α-helices not embedded inside the membrane.

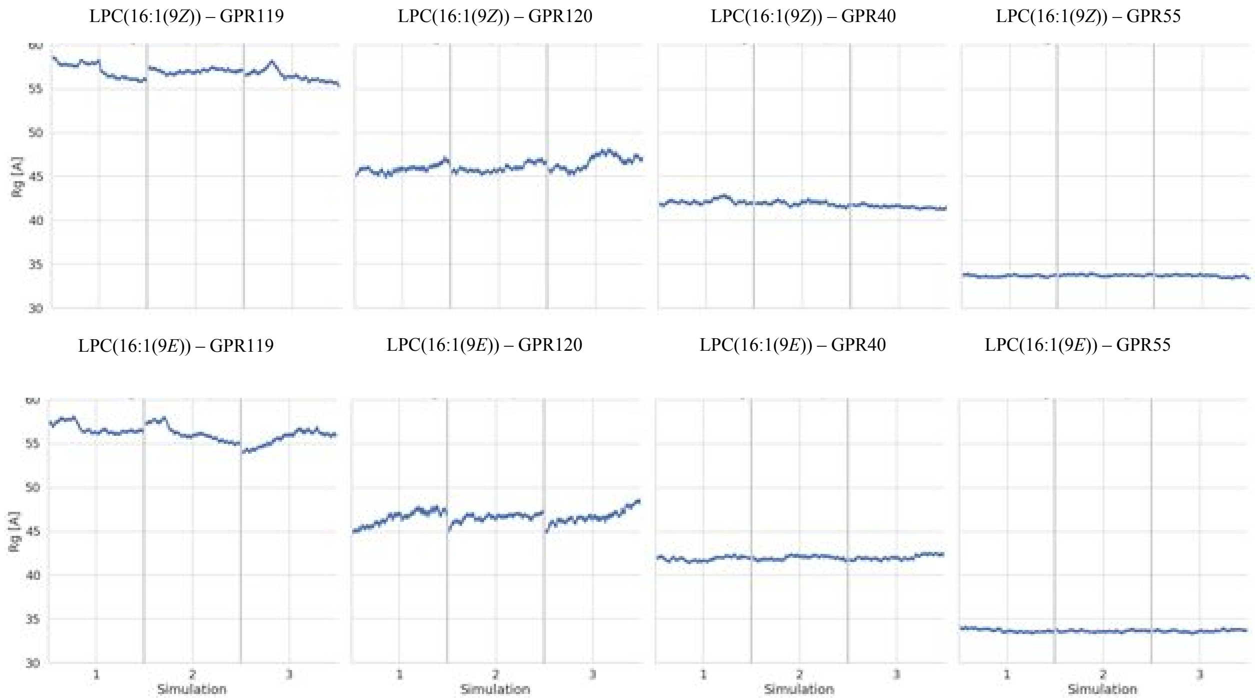

Fig S2. The radius of gyration of each receptor during simulations

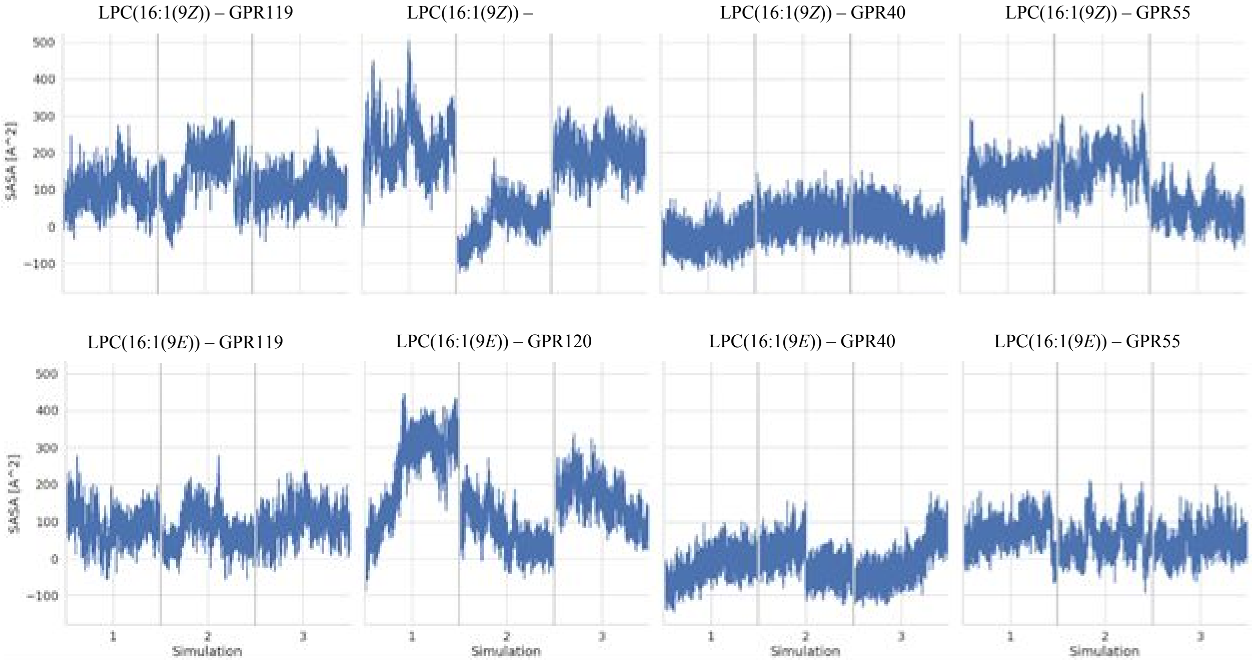

Fig S3. The solvent accessible surface area of each ligand during simulations

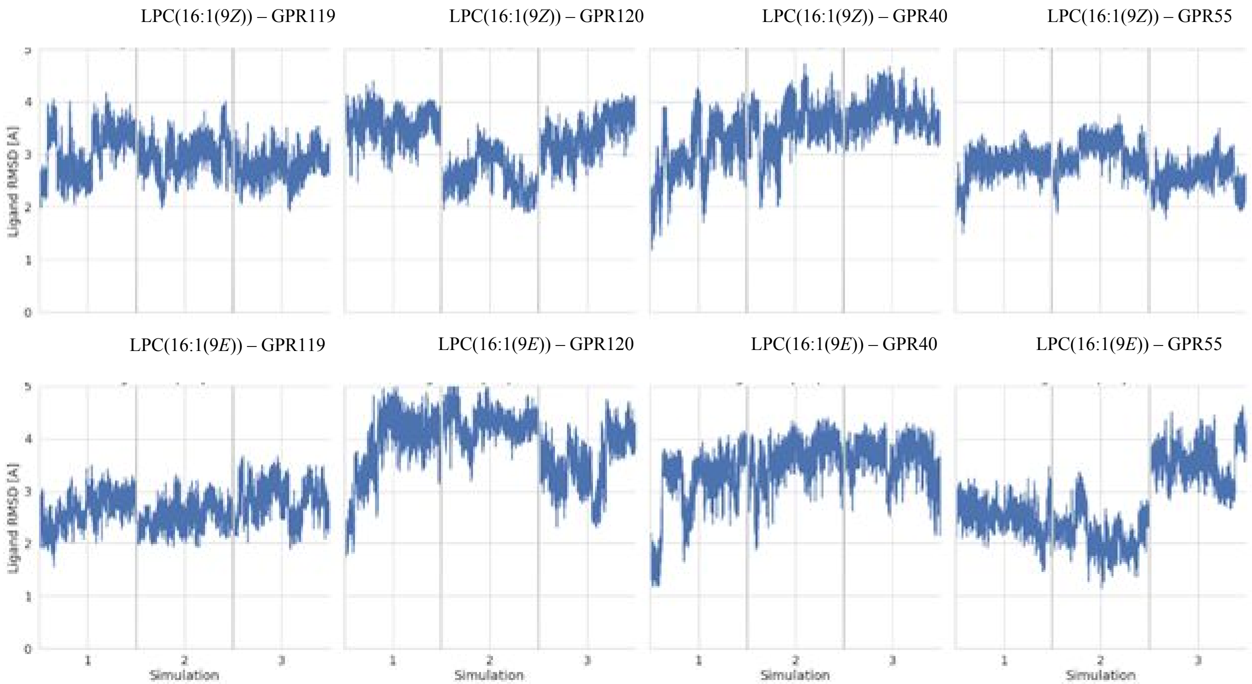

Fig S4. Root mean square deviation of ligand during simulations. High values indicate conformation change inside the binding cavity.

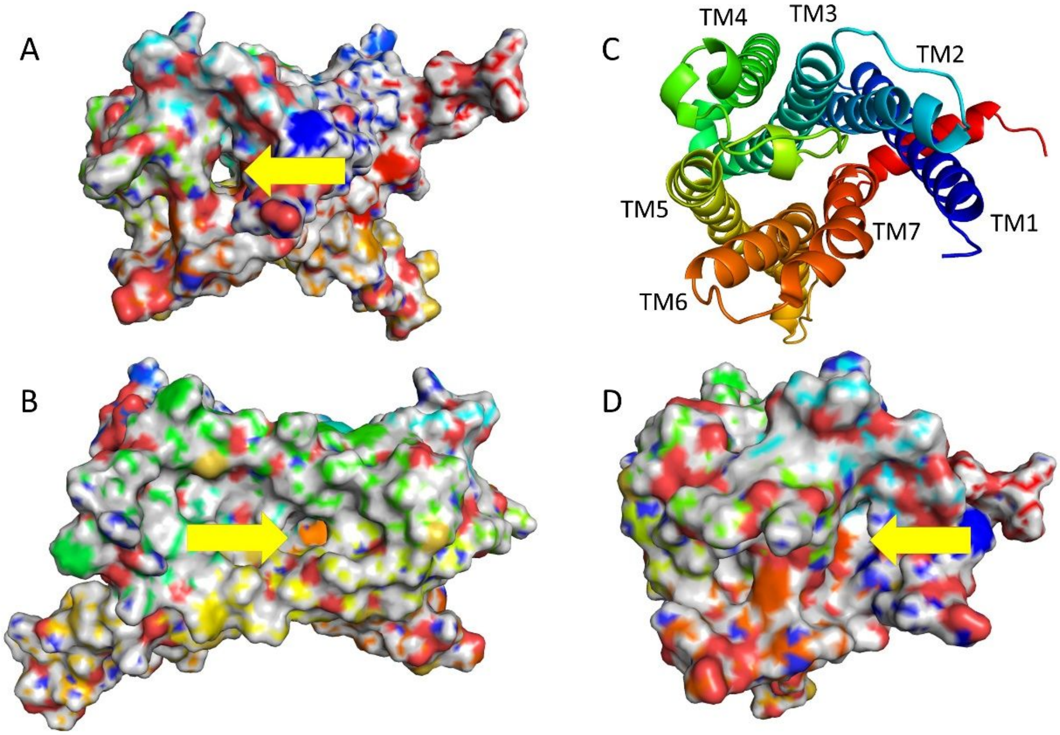

Fig S5. Structure of GPR119 from different angle with binding pocket indication (yellow arrow). A – presentation of a biding pocket cavity from the extracellular side. B – side view of GPR119 with the end of biding pocket. C – arrangement of seven transmembrane alpha helices in GPR119. D – extracellular view of GPR119 with the direction of binding pocket entrance.

Receptor	GPR40	
Ligand	LPC(16:1(9*Z*))	LPC(16:1(9*E*))
Side receptor view	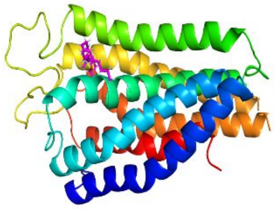	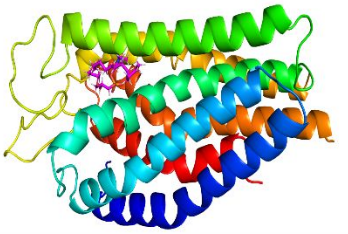
Top receptor view (extracellular side)	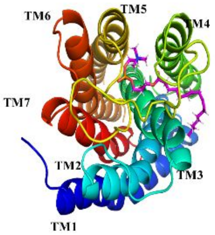	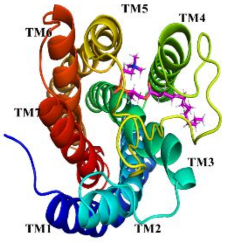
Hydrogen interactions	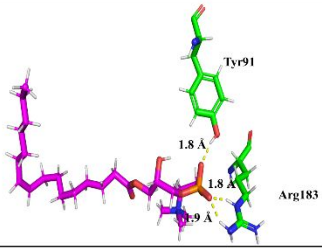	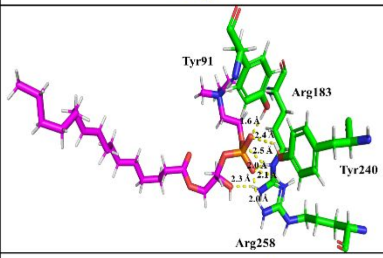
GPR119-LPC key interactions	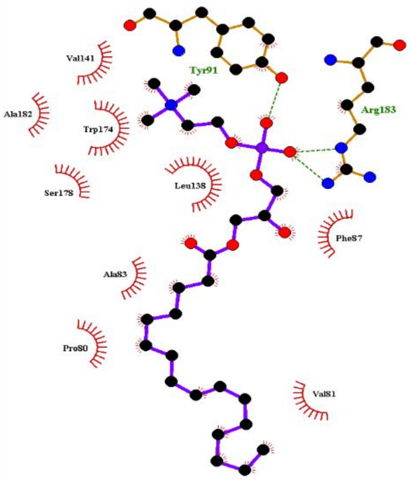	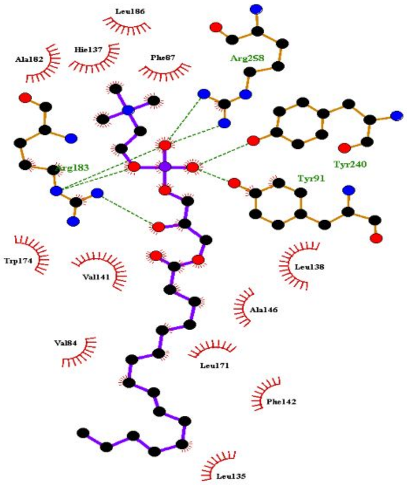
Residues involved in interactions	Hydrogen bonds: Tyr91, Arg183 (2)<br>Other interactions: Pro80, Val81, Ala83, Phe87, Leu138, Val141, Trp174, Ser178, Ala182	Hydrogen bonds: Tyr91, Arg183 (3), Tyr240, Arg258 (2)<br>Other interactions: Val84, Phe87, Leu135, Hie137, Leu138, Val141, Phe142, Ala146, Trp174, Ala182, Leu186

Fig S6. Most frequent position obtained through clustering LPC(16:1(9*Z*)) and LPC(16:1(9*E*)) inside the GPR40.

<table>
<tr><td>Receptor</td><td colspan="2">GPR55</td></tr>
<tr><td>Ligand</td><td>LPC(16:1(9Z))</td><td>LPC(16:1(9E))</td></tr>
<tr><td>Side receptor view</td><td>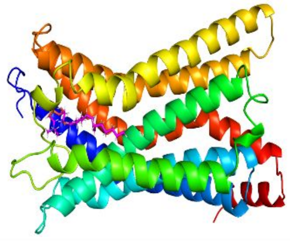</td><td>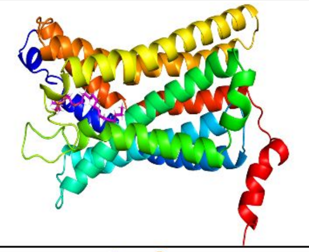</td></tr>
<tr><td>Top receptor view (extracellular side)</td><td>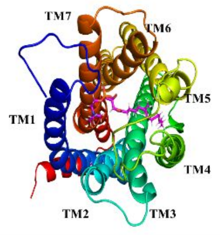
</td><td>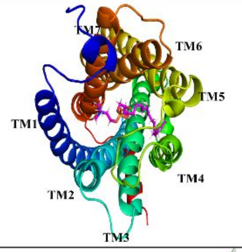
</td></tr>
<tr><td>Hydrogen interactions</td><td>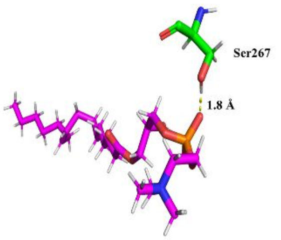
</td><td>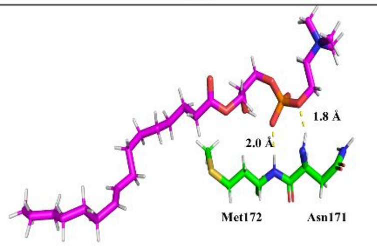
</td></tr>
<tr><td>GPR119-LPC key interactions</td><td>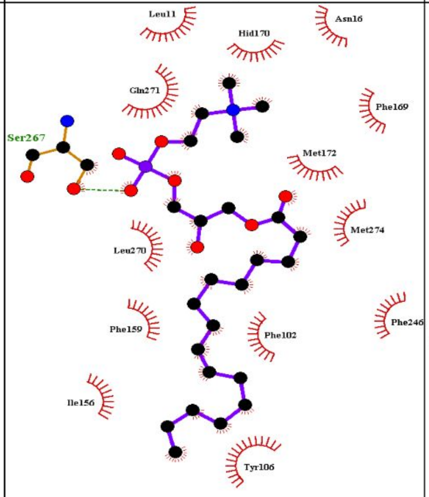
</td><td>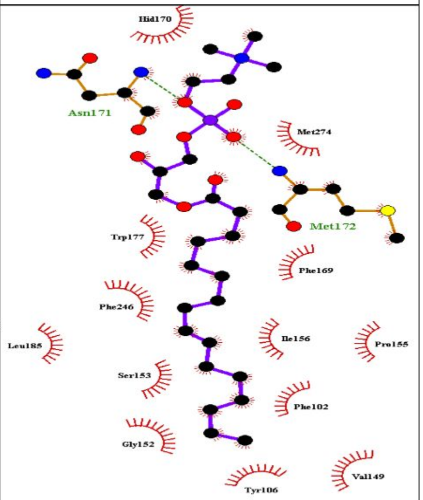
</td></tr>
<tr><td>Residues involved in interactions</td><td>Hydrogen bonds: Ser267
Other interactions: Leu11, Asn16, Phe102, Tyr106, Ile156, Phe159, Phe169, Hid170, Met172, Phe246, Leu270, Gln271, Met274</td><td>Hydrogen bonds: Asn171, Met172
Other interactions: Phe102, Tyr106, Val149, Gly152, Ser153, Pro155, Ile156, Phe169, Hid170, Trp177, Leu185, Phe246, Met274</td></tr>
</table>

Fig S7. Most frequent position obtained through clustering LPC(16:1(9*Z*)) and LPC(16:1(9*E*)) inside the GPR55.

Receptor	GPR120	
Ligand	LPC(16:1(9*Z*))	LPC(16:1(9*E*))
Side receptor view	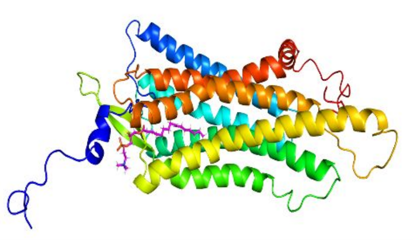	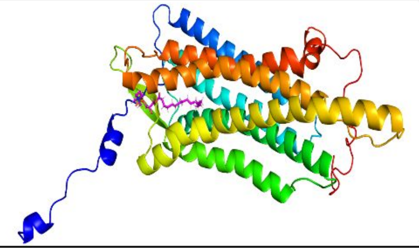
Top receptor view (extracellular side)	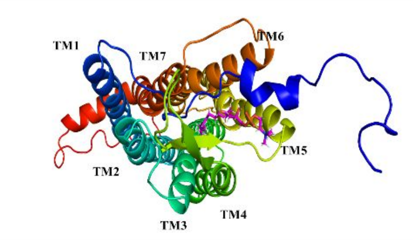	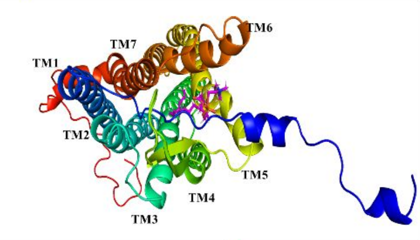
Hydrogen interactions	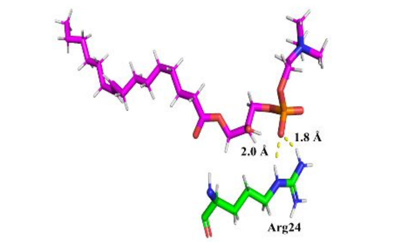	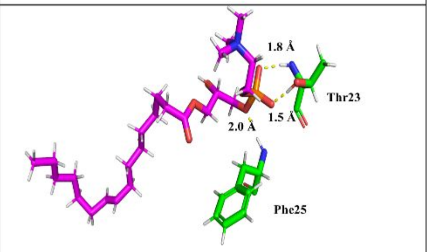
GPR119-LPC key interactions	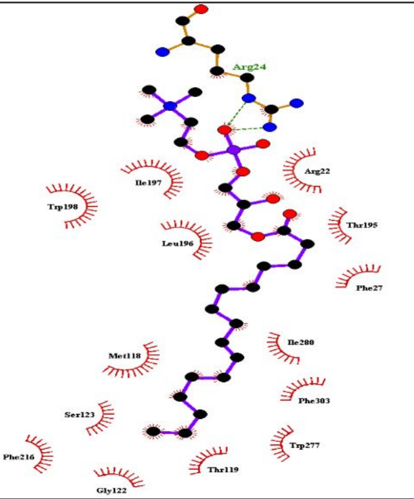	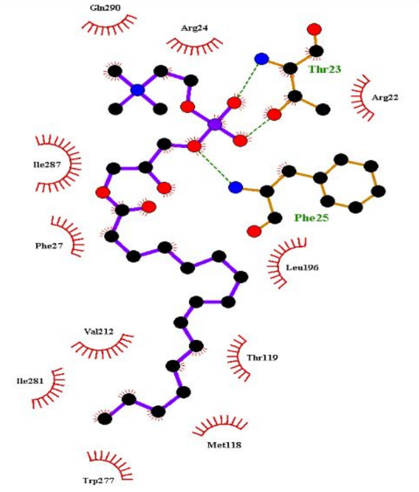
Residues involved in interactions	Hydrogen bonds: Arg24 (2)<br>Other interactions: Arg22, Phe27, Met118, Thr119, Gly122, Ser123, Thr195, Leu196, Ile197, Trp198, Phe216, Trp277, Ile280, Phe303	Hydrogen bonds: Thr23 (2), Phe25<br>Other interactions: Arg22, Arg24, Phe27, Met118, Thr119, Leu196, Val212, Trp277, Ile281, Ile287, Gln290

Fig S8. Most frequent position obtained through clustering LPC(16:1(9*Z*)) and LPC(16:1(9*E*)) inside the GPR120.

In silico mutagenesis

Models obtained after molecular dynamics was paste into ICM-Pro (ver. 3.9) software (MolsoftLLC) where implemented module to *in silico* mutagenesis was used to calculate binding energy of receptor pocket. Subsequently, all amino acids involved in ligand – protein interaction was replaced with alanine. Then new binding energy (ΔGbind mut) was calculated and compared to wild type protein binding energy (ΔGbind wt). The difference in the binding free energies was calculated with formula: ΔΔGbind = ΔGbind mut –Δgbind wt .

Mutation of Phe157 and Trp238 in both analyzed models caused the biggest change in binding energy. It leads to the conclusion that these two amino acid residues are the most important for ligand binding in the case of LPCs. Qian et al. also indicated that Trp238 is the most important residue but Phe157 was not highlighted.[7] Probably Phe157 is necessary in ligand stabilization. Also, Phe241 mutation caused reduction of binding energy in both models because of its nonpolar side chain which stabilizes ligand inside the binding pocket. For both models, Glu261 and Trp265 have significant importance in ligand-protein interaction. Moreover, the mutation of amino acids located at the entrance to the binding pocket in the GPR119-LPC(16:1(9E)) model lowers binding energy (Fig S9). This observation leads to the conclusion that all these mentioned amino acids play a crucial role in ligand binding.

Fig S9. Binding energy changes in mutants where amino acids located in receptor pocket are replaced with alanine.

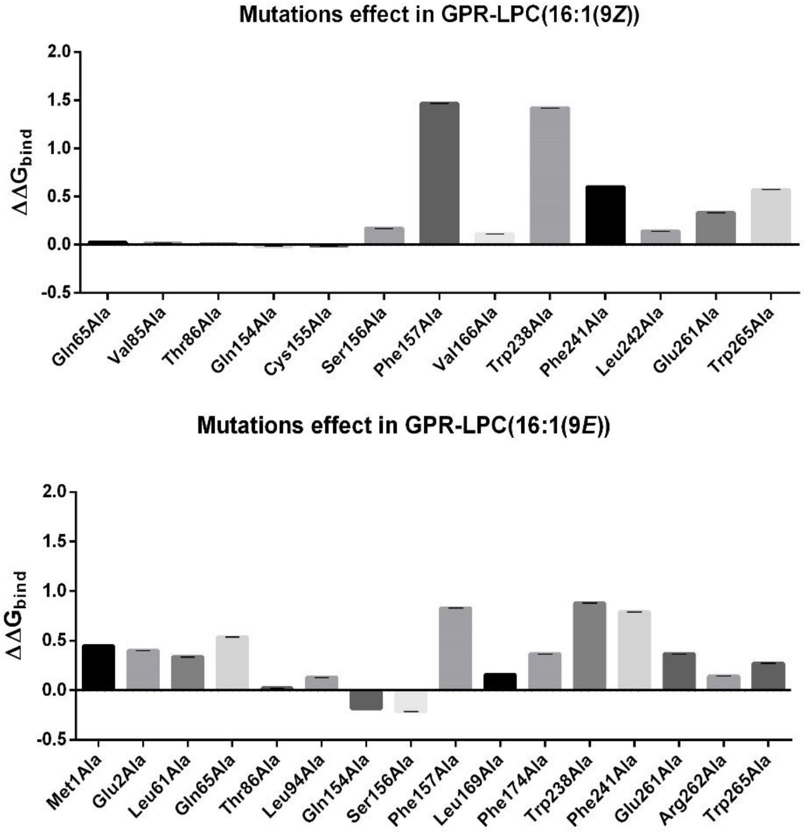

Data represent the means ± SD from at least 3 simulations.

LPC synthesis

Materials

Palmitoleic acid (*c*9 POA) ((9*Z*)-hexadecenoic acid) and palmitelaidic acid (*t*9 POA) ((9*E*)-hexadecenoic acid) were purchased from Cayman Chemical Co. *sn*-Glycero-3-phosphocholine (GPC) in the enantiomerically pure form was ordered from Bachem whereas DBTO, TEA and oxalyl chloride came from Sigma–Aldrich as well as chloroform, methanol, dichloromethane and 2-propanol.

Methods of analysis

Analytical thin-layer chromatography (TLC) was performed on Merck Kieselgel 60 F_{254} plates (0.2 mm silica gel with fluorescent indicator UV254) with mixtures of $CHCl_3/CH_3OH/H_2O$ (65:25:4, *v*/*v*/*v*). The compounds were detected by spraying the plates with 0.05% primuline solution acetone/H_2O (8:2, *v*/*v*) followed by UV (365 nm) visualization, or a solution of 10 g of $Ce(SO_4)_2$ and 20 g of phosphoromolibdenic acid in 1 L of 10% H_2SO_4 followed by heating. Column chromatography was performed on silica gel 60 with 0.1% of Ca (230–400 mesh ASTM, Merck) using a solvent mixture of $CHCl_3/CH_3OH/H_2O$ (65:25:4, *v*/*v*/*v*). All the NMR spectra were recorded on a Bruker Avance II 600 MHz spectrometer (Brüker, Billerica, MA, USA). High resolution mass spectra (HRMS) were obtained using electron spray ionization (ESI) technique on Waters ESI-Q-TOF Premier XE spectrometer. The chemical shifts were calibrated using the chloroform proton signals at 7.26 ppm in the ^1H NMR spectra and $CDCl_3$ carbon atoms at 77.0 ppm in the ^{13}C NMR spectra.

Synthesis of 1-acyl-2-hydroxy-sn-glycero-3'-phosphatidylcholines ***(2a-b)***

Lysophosphocholines **2a** and **2b** were synthesized according to the previously described procedure[8]. The crude products were purified by silica gel chromatography. Their chemical structures were confirmed by spectroscopic analysis.

1-palmitoleoyl-2-hydroxy-sn-glycero-3-phosphocholine ***(2a)***

Colourless greasy solid (49% yield, R_f 0.12); **^{1}H NMR** (600 MHz, $CDCl_3/CD_3OD$ 2:1 (v/v)), δ: 0.66 (t, J = 6.9 Hz, 3H, CH_3-16), 1.05-1.09 (m, 16H, CH_2-4, CH_2-5, CH_2-6, CH_2-7, CH_2-12, CH_2-13, CH_2-14, CH_2-15), 1.40 (m, 2H, -C**<u>H₂</u>**$CH_2C(O)$), 1.79 (m, 4H, CH_2-8, CH_2-11), 2.13 (t, J = 7.4 Hz, 2H, -C**<u>H₂</u>**C(O)), 3.03 (s, 9H, -$N(CH_3)_3$), 3.44-3.49 (2m, 3H, CH_2-β, OH), 3.69-3.79 (2m, 3H, CH_2-3′, H-2′), 3.95 (m, 2H, CH_2-1′), 4.12 (m, 2H, CH_2-α), 5.12 (2m, 2H, H-9 and H-10); **^{13}C NMR** (150 MHz, $CDCl_3/CD_3OD$ 2:1 (v/v)) δ: 13.79 (CH_3-16), 22.52 (CH_2-15), 24.76 (-**<u>C</u>**$H_2CH_2C(O)$), 27.04 and 27.08 (CH_2-8, CH_2-11), 28.85, 29.02, 29.04, 29.12, 29.61, 31.68 (C-4, C-5, C-6, C-7, C-12, C-13, C-14), 33.95 ($CH_3(CH_2)_{13}$**<u>C</u>**$H_2C(O)$), 53.97 ((-N(**<u>C</u>**$H_3)_3$), 59.52 (C-α), 64.85 (C-1′), 66.14 (C- β), 67.07 (C-3′), 68.42 (C-2′), 129.59 and 129.90 (CH-9, CH-10), 174.36 (C-1); **^{31}P NMR** (121 MHz, $CDCl_3/CD_3OD$ 2:1 (v/v)) δ: -4.22; HRMS (ESI): *m/z* calcd. for $C_{24}H_{48}NO_7P$ $[M + H]^+$ 494.3247; found 494.3263

1-palmitelaidiceoyl-*2-hydroxy-sn-glycero-3-phosphocholine* ***(2b)***

Colourless greasy solid (48% yield, R_f 0.12); **^{1}H NMR** (600 MHz, $CDCl_3/CD_3OD$ 2:1 (v/v)), δ: 0.70 (t, *J* = 7.3 Hz, 3H, CH_3-16), 1.12-1.16 (m, 18H, CH_2-3, CH_2-4, CH_2-5, CH_2-6, CH_2-7, CH_2-12, CH_2-13, CH_2-14, CH_2-15), 1.72 (m, 4H, CH_2-8, CH_2-11), 2.11 (t, *J* = 7.6 Hz, 2H, -C**<u>H₂</u>**C(O)), 3.01 (s, 9H, -$N(CH_3)_3$), 3.46 (m, 2H, CH_2-β), 3.63-3.92 (2m, 5H, CH_2-1′, CH_2-3′, H-2′), 4.08 (m, 2H, CH_2-α), 5.13 (2m, 2H, H-9 and H-10); **^{13}C NMR** (150 MHz, $CDCl_3/CD_3OD$ 2:1 (v/v)) δ: 13.78 (CH_3-16), 22.51 (CH_2-15), 24.75 (-**C**H_2CH_2C(O)), 26.08 and 27.15 (CH_2-8, CH_2-11), 28.69, 28.85, 29.02, 29.05, 29.48, 30.39, 31.63 (C-4, C-5, C-6, C-7, C-12, C-13, C-14), 33.95 ($CH_3(CH_2)_{13}$**C**H_2C(O)), 53.97 ((-N(**C**$H_3)_3$), 59.46 (C-α), 64.83 (C-1′), 66.19 (C- β), 66.94 (C-3′), 68.50 (C-2′), 130.11 and 130.39 (CH-9, CH-10), 174.43 (C-1); **^{31}P NMR** (121 MHz, $CDCl_3/CD_3OD$ 2:1 (v/v)) δ: -4.22; HRMS (ESI): *m/z* calcd. for $C_{24}H_{48}NO_7P$ $[M + H]^+$ 494.3247; found 494.3250

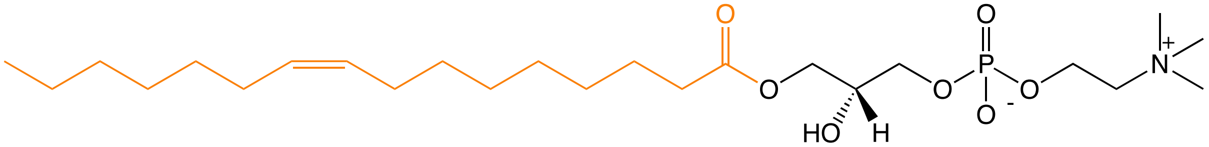

1-palmitoleoyl-2-hydroxy-sn-glycero-3-phosphocholine ***(2a)***

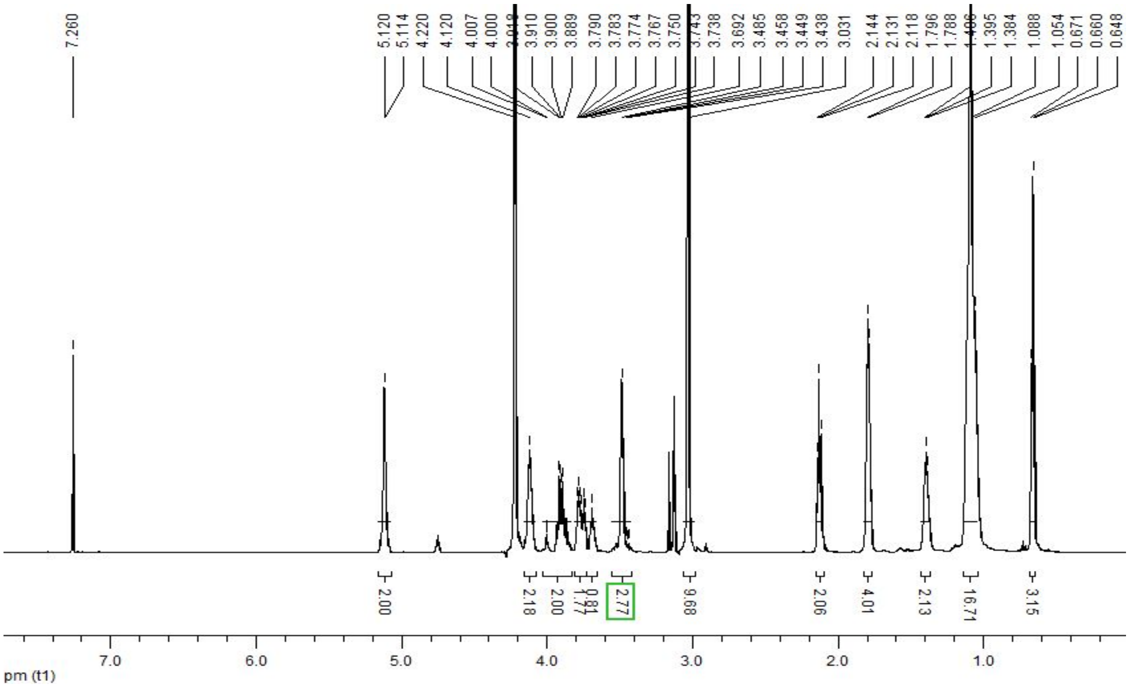

$_1$H NMR spectrum of 2a

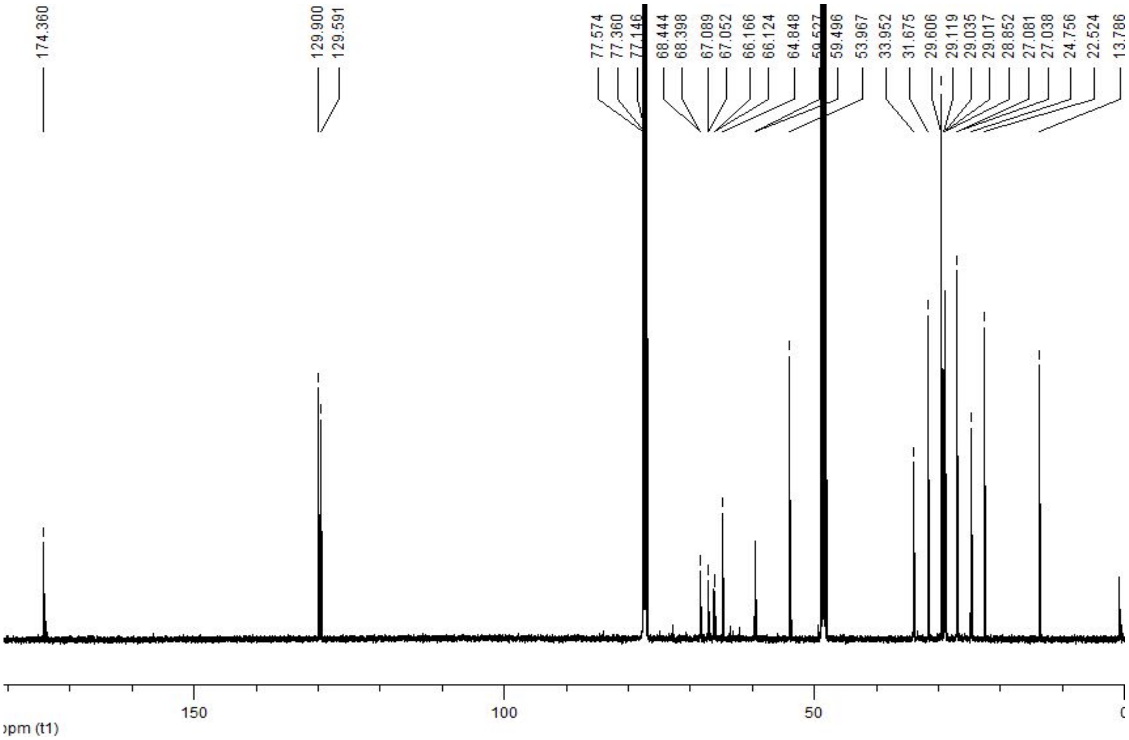

$_{13}$C NMR spectrum of 2a.

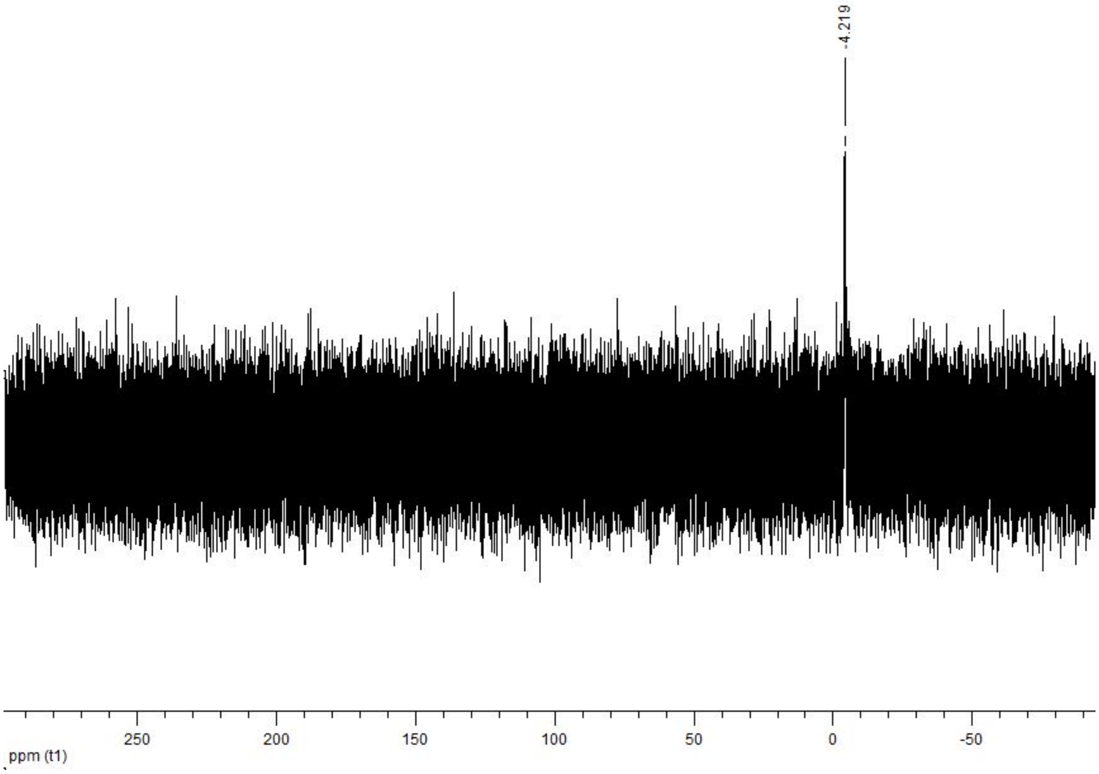

$_{31}$P NMR spectrum of 2a.
Fig S10. $_{1}$H and $_{13}$C NMR spectra and $_{31}$P NMR of 1-palmitoleoyl-2-hydroxy-sn-glycero-3-phosphocholine.

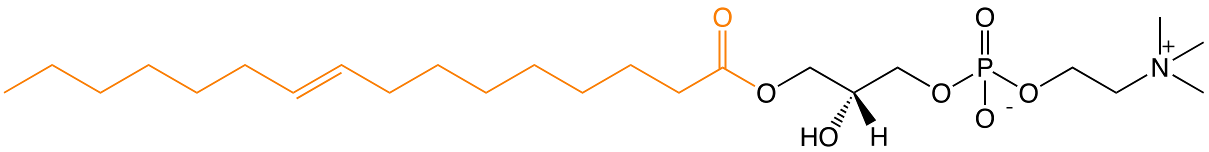

1-palmitelaidiceoyl-2-hydroxy-sn-glycero-3-phosphocholine ***(2b)***

$_1$H NMR spectrum of 2a

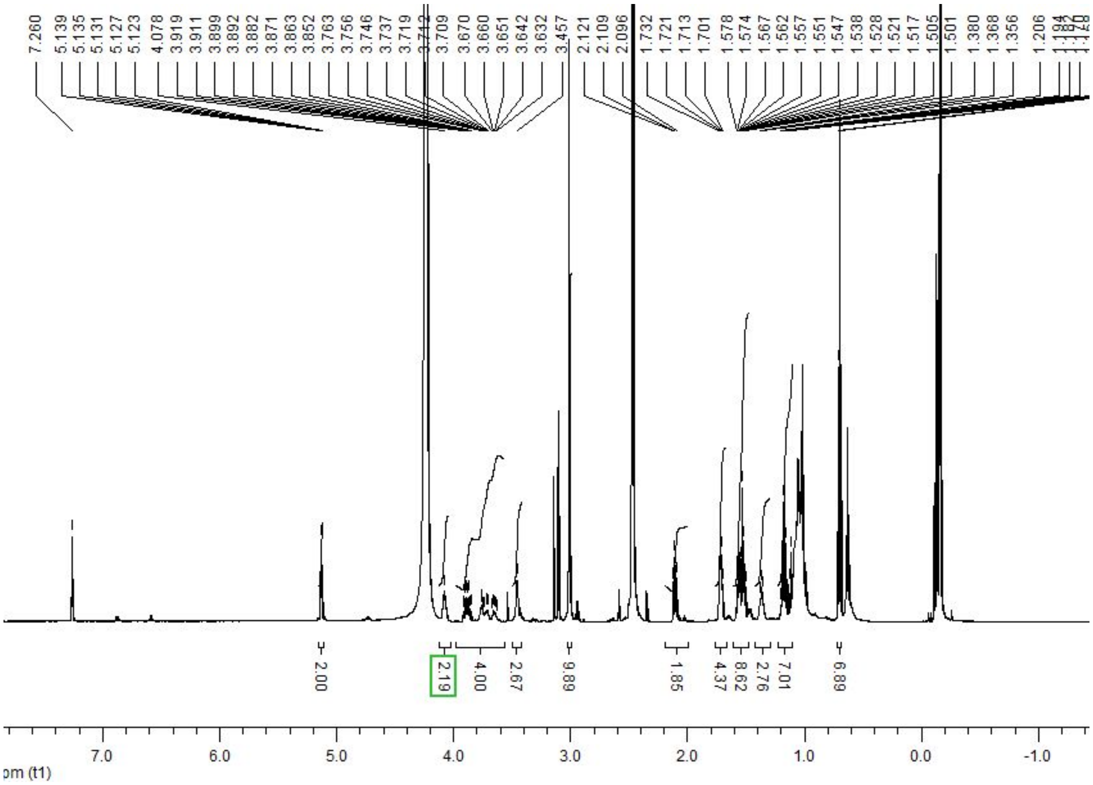

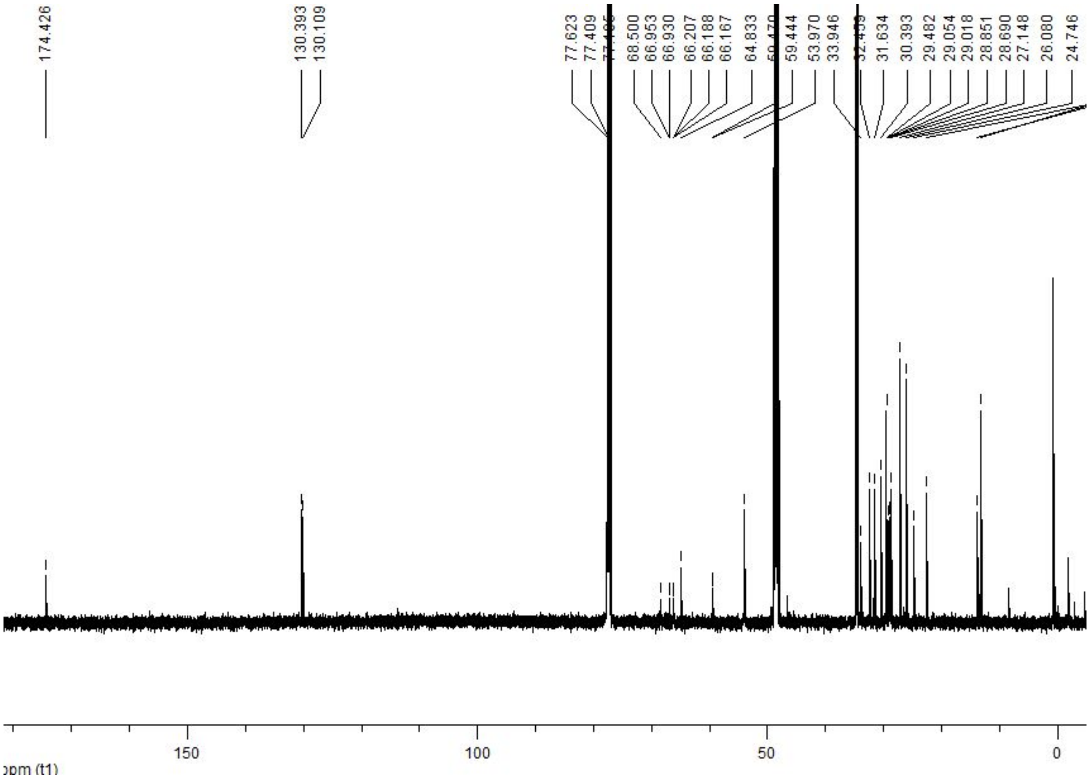

^{13}C NMR spectrum of 2a.

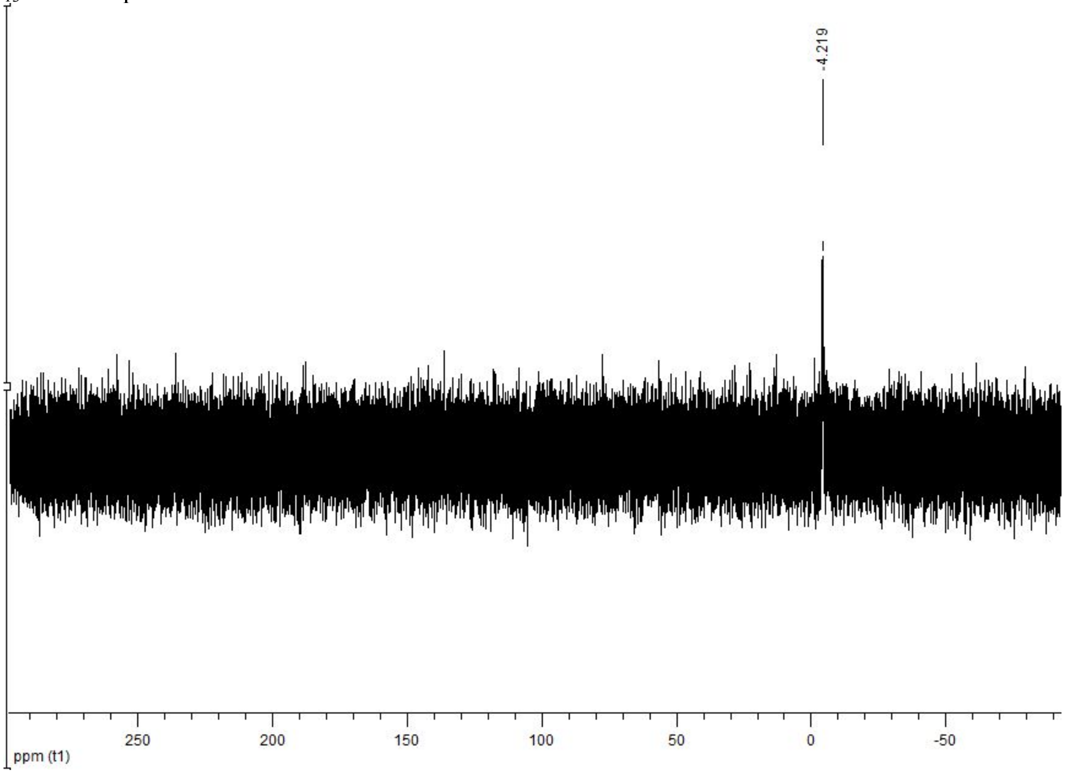

^{31}P NMR spectrum of 2a.

Fig S11. ^{1}H and ^{13}C NMR spectra and ^{31}P NMR of 1-palmitelaidiceoyl-2-hydroxy-sn-glycero-3-phosphocholine.

Cell viability assay

EndoC-βH1 cells were seeded in 96-well plates at 3 x 10^4 cells per well. After a 24-hour incubation at 37°C, the culture medium was changed to BSA-free medium in a volume of 100 µl/well. LPCs (LPC(16:0), LPC(16:1(9*Z*)) and LPC(16:1(9*E*)) were then added at final concentrations of 1 µM, 5 µM, 10 µM, 25 µM, 50 µM, and 100 µM. Cells were incubated with compounds for 24 hours, 48 hours and 72 hours at 37°C. After the specified time, PrestoBlue™ Cell Viability Reagent was added to the plates in a volume of 4 µl/well and incubated for 1 hour. Fluorescence was measured at excitation/emission = 530/620 nm. Cell viability was calculated as the percentage of test cell viability with compounds to untreated control cells.

Critical micelle concentration

To prevent ethanol or DMSO from affecting micelle formation, highly concentrated (50 times) stock solutions of LPCs in ethanol were prepared. Reaction solutions were prepared in glass tubes where DMSO/Ca5 buffer, stock solution (2%) and NileRed in DMSO (to final concentration 40 µM NileRed) were added. Then, the received solutions were mixed thoroughly and incubated in the dark at 37 °C for 30 minutes. Finally, 530/620 nm fluorescence was measured.

siRNA transfection

DharmaFECT™ Transfection Reagent II with siRNA purchased from Horizon company were used to silence *GPR119* gene. Also, positive and negative siRNA controls were used with nucleotide sequences listed below (Table S1)

Table S1. siRNA nucleotide sequences used in this study

	Name	Target sequence
1	Non-targeting siRNA (nega0tive control)	UAGCGACUAAACACAUCAA
2	Human GPR119 siRNA	UCAAAGCUCUCCGUACUGU
3	GAPDH Control siRNA (positive control)	UGGUUUACAUGUUCCAAUA

To confirm siRNA silencing, RT-qPCR was performed. EndoC-βH1 cells were seeded onto coated 12-well plates (5 x 10^5 cells per well) in 1 ml of culture medium and incubated for 24 h under optimum conditions. Then medium was substituted with a medium containing siRNA (25 nM) and transfection reagent (0,4%), without antibiotics according to the transfection protocol provided by the producer. After 48 hours of incubation, total RNA was isolated from the cells with an Universal RNA Purification kit (EURx, Poland). Subsequently, RNA was transcribed into cDNA with NG dART RT kit (EURx, Poland) and qPCR was performed (SG qPCR Master Mix, EURx, Poland) in in CFX96 Touch Real-Time PCR Detection System. GAPDH primers: forward: 5'AGGGCTGCTTTTAACTCTGGT3', reverse: 5'CCCCACTTGATTTTGGAGGGA3'; GPR119 primers: forward: 5'CTCCCTCATCATTGCTACTAA3' reverse: 5'ACAGCCAGATTCAAGGTG3'. Relative expression was calculated as a difference between cycle threshold (CT) of analyzed gene and control calibrator *GAPDH*, and control cells without siRNA stimulation. Results are presented below (Table S2)

Table S2. RT-qPCR analysis of GPR119 knockdown using siRNA.

siRNA	Relative expression ± SD (Livak's method)
Non-targeting siRNA	1.05 ± 0.17
Human GPR119 siRNA	0.77 ± 0.17
GAPDH Control siRNA	0.36 ± 0.01

Calcium flux measurements

To perform intracellular Ca^{2+} measurements ($[Ca^{2+}]_i$), EndoC-βH1 cells were seeded onto coated 96-well plates (4 x 10^4 cells per well) in 100 µl of culture medium and incubated for 24 h under optimum conditions. On the following day, the culture medium was substituted with Ca5 buffer containing 2 mM or 20 mM glucose and based on the given protocol from Screen QuestTM Fluo-8 No Wash Calcium Assay Kit, $[Ca^{2+}]_i$ was measured. Additionally, to examine plausible membrane permeability caused by perused compounds, propidium iodide (PI) was added (final concentration of 1 µg/ml) just before running the experiment in the microplate reader. Changes in fluorescence measurements for calcium flux (Ex/Em 490/520 nm) and PI intercalation (Ex/Em 535/617 nm) were monitored upon the addition of LPCs. Fluorescence measurements were corrected with reference to background fluorescence.

Glucose stimulated insulin secretion (GSIS)

EndoC-βH1 cells were seeded onto a coated 24-well plate at the density of 2.5 × 10^5 cells/well in 1 ml of culture medium and incubated at 37 °C to reach 80-90% confluency. Next, cells were washed with calcium buffer Ca5 (25 mM HEPES, 125 mM NaCl, 6 mM KCl, 1,2 mM $MgCl_2$:6H_2O, 1,3 mM $CaCl_2$:2H_2O; pH 7,4) and incubated with the same buffer supplemented with 2 mM glucose for 1 hour at 37 °C. The buffer was refreshed with Ca5 buffer containing 2 mM glucose again. 5 µM of tested samples (LPC(16:0), LPC(16:1(9*Z*)) and LPC(16:1(9*E*)) purely or in combination with 2 µM of GPR40, GPR55, GPR119 and GPR120 antagonists (DC, CID, C8, and AH respectively) were also added. Cells were incubated for 30 minutes at 37 °C. 200 µl of sample was collected and mixed with BSA at the final concentration of 0.1%. The buffer was then substituted with a new portion of Ca5 buffer supplemented with 20 mM glucose and tested compounds and/or antagonists were added at the same concentrations. Cells were incubated for 30 minutes once again. 200 µl sample was collected again and mixed with BSA. Further on, the adherent cells were lysed with the addition of 0.1 M HCl (100 µl/well). Collected samples were used to evaluate insulin content via competitive enzyme-linked immunosorbent (ELISA) assay and the amount of secreted insulin was normalized to the protein content of the respective cell lysates measured by Bradford Protein assay.

siRNA transfection preceding GSIS

To find out if *GPR119* gene silencing affects GSIS, EndoC-βH1 cells were seeded onto a coated 24-well plate at the density of 1 × 10^5 cells/well in 1 ml of culture medium and incubated at 37 °C for 24 hours. Then the medium was

substituted with a medium containing siRNA (25 nM) and transfection reagent (0,4%), without antibiotics according to the transfection protocol provided by the producer. After 48 hours GSIS procedure proceeded as described above.

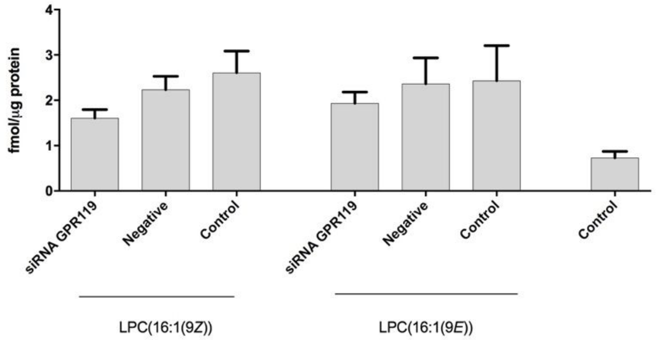

Figure S11. Insulin secretion by EndoC-βH1 cells after 48 hours of transfection with siRNA directed to silence *GPR119* expression.

cAMP synthesis measurements

Analysis of stimulated cAMP synthesis was performed using a Cyclic AMP ELISA Kit based on the supplier's protocol. EndoC-βH1 cells were seeded onto 6-well plates (2 x 10^6 cells per well) and cultivated for 48 h under optimum conditions. Subsequently, the standard culture medium was replaced with Ca5 buffer with 2 mM glucose and incubated for 1 h under standard conditions. Next, the buffer was changed to a fresh one containing 20 mM glucose. Fatty acids, with a final concentration of 10 µM, and IBMX, with a final concentration of 1mM, were also added. Cells were again incubated for 30 minutes under the same conditions. Accumulated cAMP concentration was measured in cell lysates and recalculated with respect to the protein content of cells, which was previously measured using Bradford Protein assay.

Safety statement

No unexpected or unusually high safety hazards were encountered.

References

(1) McNutt, A. T.; Francoeur, P.; Aggarwal, R.; Masuda, T.; Meli, R.; Ragoza, M.; Sunseri, J.; Koes, D. R. GNINA 1.0: molecular docking with deep learning. *J. Cheminform.* **2021**, *13* (1), 1–20. https://doi.org/10.1186/S13321-021-00522-2/FIGURES/13.

(2) Case, D. .; Kollman, P. .; Cheatham, T. .; Merz, K. .; Simmerling, C. .; Luo, R. .; Walker, R. .; Kollman, P. Amber 2020 . 2020.

(3) Korkus, E.; Dąbrowski, G.; Szustak, M.; Czaplicki, S.; Madaj, R.; Chworoś, A.; Koziołkiewicz, M.; Konopka, I.; Gendaszewska-Darmach, E. Evaluation of the anti-diabetic activity of sea buckthorn pulp oils prepared with different extraction methods in human islet EndoC-betaH1 cells. *NFS J.* **2022**, *27*, 54–66. https://doi.org/10.1016/J.NFS.2022.05.002.

(4) Jumper, J.; Evans, R.; Pritzel, A.; Green, T.; Figurnov, M.; Ronneberger, O.; Tunyasuvunakool, K.; Bates, R.; Žídek, A.; Potapenko, A.; Bridgland, A.; Meyer, C.; Kohl, S. A. A.; Ballard, A. J.; Cowie, A.; Romera-Paredes, B.; Nikolov, S.; Jain, R.; Adler, J.; Back, T.; Petersen, S.; Reiman, D.; Clancy, E.; Zielinski, M.; Steinegger, M.; Pacholska, M.; Berghammer, T.; Bodenstein, S.; Silver, D.; Vinyals, O.; Senior, A. W.; Kavukcuoglu, K.; Kohli, P.; Hassabis, D. Highly accurate protein structure prediction with AlphaFold. *Nat. 2021 5967873* **2021**, *596* (7873), 583–589. https://doi.org/10.1038/s41586-021-03819-2.

(5) Li, Y.; Liu, J.; Gumbart, J. C. Preparing Membrane Proteins for Simulation Using CHARMM-GUI. *Methods Mol. Biol.* **2021**, *2302*, 237–251. https://doi.org/10.1007/978-1-0716-1394-8_13/COVER.
(6) Adasme, M. F.; Linnemann, K. L.; Bolz, S. N.; Kaiser, F.; Salentin, S.; Haupt, V. J.; Schroeder, M. PLIP 2021: expanding the scope of the protein–ligand interaction profiler to DNA and RNA. *Nucleic Acids Res.* **2021**, *49* (W1), W530–W534. https://doi.org/10.1093/NAR/GKAB294.
(7) Qian, Y., Wang, J., Yang, L., Liu, Y., Wang, L., Liu, W., Lin, Y., Yang, H., Ma, L., Ye, S., Wu, S., & Qiao, A. (2022). Activation and signaling mechanism revealed by GPR119-Gs complex structures. Nature Communications 2022 13:1, 13(1), 1–10. https://doi.org/10.1038/s41467-022-34696-6
(8) Czarnecka, M.; Switalska, M.; Wietrzyk, J.; Maciejewska, G.; Gliszczyńska, A. Synthesis and biological evaluation of phosphatidylcholines with cinnamic and 3-methoxycinnamic acids with potent antiproliferative activity. *RSC Adv.* **2018**, *8* (62), 35744–35752. https://doi.org/10.1039/C8RA07002D.
